# Synthesis, Antimicrobial Activity, Structure-Activity Relationship, and Molecular Docking Studies of Indole Diketopiperazine Alkaloids

**DOI:** 10.3389/fchem.2019.00837

**Published:** 2019-11-29

**Authors:** Bin Jia, Yang-min Ma, Bin Liu, Pu Chen, Yan Hu, Rui Zhang

**Affiliations:** ^1^Shaanxi Key Laboratory of Chemical Additives for Industry, College of Chemistry and Chemical Engineering, Shaanxi University of Science and Technology, Xi'an, China; ^2^School of Pharmacy, Shaanxi Institute of International Trade and Commerce, Xi'an, China; ^3^Collaborative Innovation Center of Green Manufacturing Technology for Traditional Chinese Medicine in Shaanxi Province, Xi'an, China; ^4^School of Arts and Sciences, Shaanxi University of Science and Technology, Xi'an, China

**Keywords:** indole diketopiperazine alkaloids, antimicrobial activity, structure-activity relationship, molecular docking, *Escherichia coli* FabH

## Abstract

Strategies for the synthesis of indole diketopiperazine alkaloids (indole DKPs) have been described and involve three analogs of indole DKPs. The antimicrobial activity and structure-activity relationship (SAR) of 24 indole DKPs were explored. Compounds **3b** and **3c** were found to be the most active, with minimum inhibitory concentrations (MIC) values in the range of 0.94–3.87 μM (0.39–1.56 μg/mL) against the four tested bacteria (*Staphylococcus aureus, Bacillus subtilis, Pseudomonas aeruginosa*, and *Escherichia coli*). Furthermore, compounds **4a** and **4b** displayed broad-spectrum antimicrobial activity with MIC values of 1.10–36.9 μM (0.39–12.5 μg/mL) against all tested bacteria and plant pathogenic fungi (*Colletotrichum gloeosporioides, Valsa mali, Alternaria alternata* and *Alternaria brassicae*). According to the *in silico* study, compounds **3c** showed significant binding affinity to the FabH protein from *Escherichia coli*, which has been identified as the key target enzyme of fatty acid synthesis (FAS) in bacteria. Therefore, these compounds are not only promising new antibacterial agents but also potential FabH inhibitors.

## Introduction

**Figure 1:**
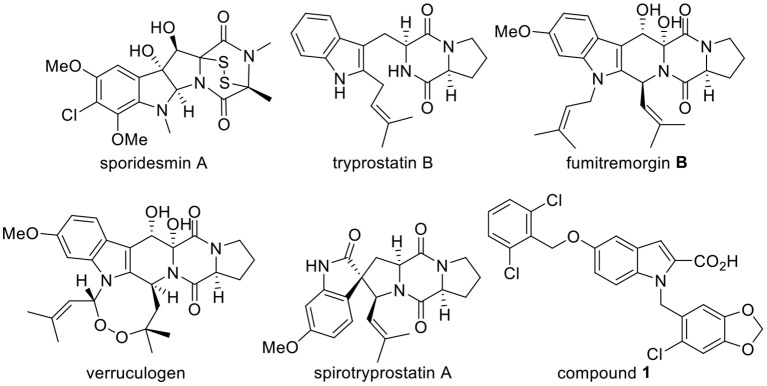
Structures of indole DKPs derivatives and FabH inhibitor compound **1**.

Indole diketopiperazine alkaloids (indole DKPs) are comprised of two bioactive heterocyclic cores, an indole and a diketopiperazine. Indole DKPs are secondary metabolites of microorganisms that are widely distributed in filamentous fungi, including *Aspergillus, Penicillium, Pestalotiopsis*, and *Chromocleista* (Ma et al., 2016). These compounds have attracted significant attention from chemical and biological researchers over the years due to their intriguing structure and wide range of biological activities. These compounds show a variety of biological activities, such as antimicrobial (Zheng et al., 2007; Sun et al., 2011; Ebead et al., 2012; El-Gendy Bel and Rateb, 2015; Yu et al., 2018), anticancer (Isham et al., 2007; Tsukamoto et al., 2008), anti-inflammatory (Liu et al., 2018), antioxidant (Zhong et al., 2019), and insecticidal (De Guzman and Gloer, 1992; Dong et al., 2005; Hayashi, 2005; Rateb et al., 2013; An et al., 2014) activities. For example, the natural product sporidesmin A (Figure 1) has good antimicrobial activity that is comparable to the well-known broad-spectrum antifungal and antibacterial agent ciclopirox (Sun et al., 2011). Tryprostatin B (Figure 1) is a fungal inhibitors of mammalian cell cycle progression at the G2/M transition (Tong Gan, 1997). Fumitremorgin B and verruculogen (Figure 1) are potential growth inhibitors of the parasites *Trypanosoma brucei brucei* and *Leishmania donovani* (Rateb et al., 2013). Although natural products of microbial origin that exist widely in nature have been proven to be effective and inherently biodegradable due to the complexity of their structures (Tareq et al., 2013), their natural abundance is scarce. Fortunately, the synthesis of indole DKPs has attracted attention from chemists. Various synthetic methods of these compounds have been explored, such as the synthesis of spirotryprostatin A (Figure 1) (Cheng et al., 2011; Kitahara et al., 2014). Moreover, developing new, harmless antimicrobial agents for foods and plants that are derived from natural compounds has attracted widespread attention (Zhao et al., 2017; Alejo-Armijo et al., 2018). Therefore, it is desirable to find active natural lead compounds from indole DKPs.

In our previous studies, 13 indole DKPs were isolated from the endophytic fungus *Aspergillus tamarii* in *Ficus carica* L., and all of the isolated indole DKPs showed potential antibacterial, antifungal and anti-phytopathogenic activities (Zhang, 2012; Zhang et al., 2012). Among these compounds, verruculogen (Figure 1) showed the best activity against the tested bacteria (*Staphylococcus aureus, Bacillus subtilis, Escherichia coli*, and *Pseudomonas aeruginosa*) with MIC values of 1.56–3.13 μg/mL. However, verruculogen cannot be considered an appropriate antimicrobial agent candidate because it causes tremors in animals. Therefore, it is essential to synthesize a series of indole DKPs for research on their biological activities. To our knowledge, only a few reports have thoroughly studied the antimicrobial activity and mechanism of indole DKPs. Therefore, it is necessary to conduct an in-depth study on these compounds.

FabH plays an essential and regulatory role in bacterial fatty acid synthesis (FAS). FabH also plays a key regulatory role in the entire biosynthetic pathway in the initiation of the fatty acid chain elongation cycle (Heatha et al., 2001). FabH is also essential for bacterial survival and for the universal processes of cell membrane generation and maintenance (Alhamadsheh et al., 2007a). In addition, FabH has been identified as the key target enzyme of FAS in bacteria and exists prevalently in a large number of clinical pathogens, such as gram-positive bacteria, gram-negative bacteria, and mycobacteria (Alhamadsheh et al., 2007b). Further research has also shown that FabH proteins are highly conserved at the gene sequence and structural level in both gram-positive and gram-negative bacteria, while no significant homologous with human tissue proteins exist. Moreover, the residues that comprise the active site are essentially invariant among gram-positive and gram-negative organisms (Wakil, 1989). Moreover, it has been reported that the mode of antimicrobial action is likely due to the inactivation of essential enzymes (Pisoschi et al., 2018). These attributes suggest that inhibitors of FabH could be potential antimicrobial agents (Nie et al., 2005). Although there are many enzymes in FAS, FabH is the most well-studied bacterial enzyme, having been cloned and characterized from a number of bacterial species (Khandekar et al., 2003). Additionally, computer docking techniques play an important role in the drug design and discovery, as well as in mechanistic studies by placing a molecule into the binding site of the target macromolecule in a non-covalent fashion (Abdel-Aziz et al., 2011). GlaxoSmithKline identified indole analog compound **1** (Figure 1) to be a potent inhibitor of *Streptococcus pneumoniae* FabH through high-throughput screening (Daines et al., 2003). This encouraging result excited us to determine the binding mode of indole DKPs with FabH via docking simulations for a better understanding of the drug-receptor interaction.

We obtained mimics of fumitremorgin B and spirotryprostatin A (Figure 1) in previous studies. In order to broaden the knowledge of the structural features that could influence the antimicrobial activities of indole DKPs, herein, we (1) designed a set of indole DKPs analogs as tryprostatin B mimics, (2) evaluated the antimicrobial activity of 24 indole DKPs and discussed the preliminary structure-activity relationship (SAR), and (3) performed docking simulations to give a prediction of the binding mode between the indole DKPs and *E. coli* FabH.

## Materials and Methods

### Chemicals and Instruments

Starting materials and all other reagents were purchased from Aladdin Chemical (Shanghai, China). All solvents were of analytical grade and purchased from Tianjin Hongyan Chemical Reagents Factory (Tianjin, China). Silica gel for column chromatography (200–300 mesh) was purchased from Tsingtao Haiyang Silica Gel Desiccant Factory (Tsingtao, China). Solvents were dried according to standard procedures. Nuclear magnetic resonance spectra (NMR) were performed on a Bruker Avance 400 instrument (^1^H NMR at 400 MHz, ^13^C NMR at 100 MHz). High-resolution mass spectra were acquired on a Bruker Daltonics MicrOTof-Q II mass spectrometer.

### Synthesis

**Scheme 1:**
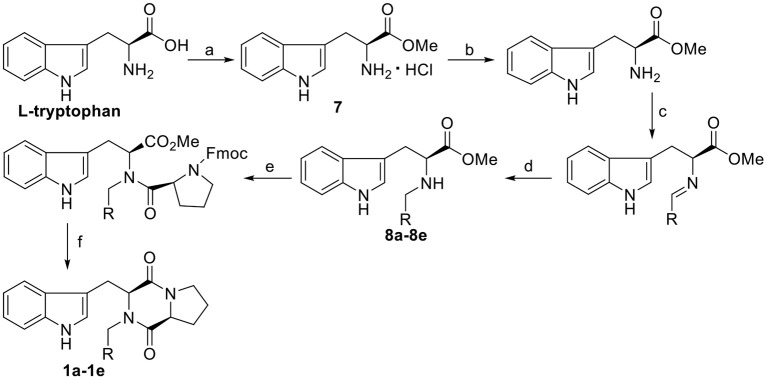
Synthesis route of the target compounds **1a−1e**. Reaction conditions: (a) SOCl_2_, CH_3_OH; (b) TEA, CH_3_OH; (c) CH(OCH_3_)_3_, CH_3_OH, R-CHO; (d) NaBH(OAc)_3_, CH_3_OH; (e) Fmoc-*L*-Pro-Cl, TEA, DCM/Na_2_CO_3_(aq); (f) morpholine, DCM.

The synthetic routes of target compounds **1a−1e** are outlined in Scheme 1.

### General Procedure for the Synthesis of Compounds 1a−1e

To a solution of Fmoc-L-Pro-Cl (3.89 g, 11 mmol) in CH_2_Cl_2_ (20 mL), the corresponding *N-*substituted L-tryptophan methyl ester (**8a−8e**) (see the Supporting Information for the synthetic procedure) (10 mmol) was added dropwise at room temperature, and the resulting reaction system was stirred for 5 min, followed by the addition of Na_2_CO_3_ (1 mol/L, 10 mL). The mixture was stirred at room temperature for 1 h, and then the organic phase was separated. To this solution, morpholine (50%, 6 mL) was added. After stirring at room temperature for 3 h, the solvent was evaporated, and the residue was purified by column chromatography with dichloromethane/ethyl acetate (v/v = 10:1) to afford the desired products.

**(3*S*,8a*S*)-3-((1*H*-indol-3-yl)methyl)-2-benzylhexahydropyr rolo[1,2-*a*]pyrazine-1,4-dione**, **1a**: yield, 55.7%; white solid; mp 251–253°C. ^1^H NMR (400 MHz, CDCl_3_): δ 8.41 (br, 1H), 7.64 (d, *J* = 8.0 Hz, 1H), 7.38–7.33 (m, 6H), 7.20 (t, *J* = 8.0 Hz, 1H), 7.12 (t, *J* = 8.0 Hz, 1H), 6.98 (d, *J* = 4.0 Hz, 1H), 5.77 (d, *J* = 16.0 Hz, 1H), 4.23 (s, 1H), 4.05 (d, *J* = 8.0 Hz, 1H), 3.80 (dd, *J* = 8.0, 4.0 Hz, 1H), 3.65 (dd, *J* = 12.0, 4.0 Hz, 1H), 3.45 (m, 1H), 3.36 (dd, *J* = 12.0, 4.0 Hz, 1H), 2.79 (td, *J* = 8.0, 4.0 Hz, 1H), 1.80 (m, 1H), 1.37 (m, 1H), 0.79 (m, 1H), −0.10 (m, 1H). ^13^C NMR (100 MHz, CDCl_3_): δ 166.4, 164.4, 135.7, 135.4, 129.0, 128.5, 128.2, 127.4, 123.9, 122.4, 119.8, 119.7, 110.9, 108.6, 59.5, 59.6, 46.0, 44.5, 28.3, 26.6, 20.6. HRMS (ESI) *m*/*z* calculated for C_23_H_24_N_3_O_2_ [M+H]^+^ 374.1869, found: 374.1864.

**(3*S*,8a*S*)-3-((1*H*-indol-3-yl)methyl)-2-(4-methoxybenzyl) hexahydropyrrolo[1,2-*a*]pyrazine-1,4-dione**, **1b**: yield, 66.3%; white solid; mp 157–159°C. ^1^H NMR (400 MHz, CDCl_3_): δ 9.01–8.54 (m, 1H), 7.64 (d, *J* = 7.9 Hz, 1H), 7.41–7.31 (m, 1H), 7.30 (s, 1H), 7.28 (s, 1H), 7.23–7.13 (m, 1H), 7.15–7.05 (m, 1H), 7.00–6.87 (m, 3H), 5.70 (d, *J* = 14.5 Hz, 1H), 4.22 (t, *J* = 3.3 Hz, 1H), 4.01 (d, *J* = 14.5 Hz, 1H), 3.84 (d, *J* = 1.3 Hz, 3H), 3.78 (dd, *J* = 11.7, 6.1 Hz, 1H), 3.65 (dd, *J* = 14.9, 2.5 Hz, 1H), 3.51–3.27 (m, 2H), 2.94 (td, *J* = 11.0, 4.6 Hz, 1H), 1.85–1.57 (m, 1H), 1.44–1.16 (m, 1H), 0.92–0.52 (m, 1H), −0.09 to −0.30 (m, 1H). ^13^C NMR (100 MHz, CDCl_3_): δ 166.04, 164.46, 136.73, 129.97, 128.15, 127.36, 119.66, 114.37, 109.82, 107.80, 59.25, 55.36, 45.48, 44.43, 28.59, 26.65, 20.68. HRMS (ESI) *m*/*z* calculated for C_24_H_26_N_3_O_3_ [M+H]^+^ 404.1974, found: 404.1969.

**(3*S*,8a*S*)-3-((1*H*-indol-3-yl)methyl)-2-cinnamylhexahydr opyrrolo[1,2-*a*]pyrazine-1,4-dione**, **1c**: yield, 49.4%; white solid; mp 181–183°C. ^1^H NMR (400 MHz, CDCl_3_) δ: 8.64 (br, 1H), 7.67 (d, *J* = 8.0 Hz, 1H), 7.42–7.28 (m, 6H), 7.18 (t, *J* = 8.0 Hz, 1H), 7.11 (t, *J* = 8.0 Hz, 1H), 6.70 (d, *J* = 16.0 Hz, 1H), 6.22 (m, 1H), 5.14 (dd, *J* = 8.0 Hz, 1H), 4.45 (s, 1H), 3.81–3.68 (m, 3H), 3.46 (m, 1H), 3.34 (dd, *J* = 12.0 Hz, 4.0 Hz, 1H), 2.96 (td, *J* = 8.0 Hz, 4.0 Hz, 1H), 1.75 (m, 1H), 1.35 (m, 1H), 0.76 (m, 1H), −0.13 (m, 1H). ^13^C NMR (100 MHz, CDCl_3_): δ 166.2, 164.4, 136.1, 135.8, 134.8, 128.7, 128.2, 127.5, 126.5, 124.0, 122.7, 122.4, 119.7, 119.6, 111.0, 108.4, 60.0, 59.3, 45.2, 44.5, 28.2, 27.0, 20.6. HRMS (ESI) *m*/*z* calculated for C_25_H_26_N_3_O_2_ [M+H]^+^ 400.2025, found: 400.2019.

**(3*S*,8a*S*)-3-((1*H*-indol-3-yl)methyl)-2-(furan-2-ylmethyl) hexahydropyrrolo[1,2-*a*]pyrazine-1,4-dione**, **1d**: yield, 41.5%; white solid; mp 169–171°C. ^1^H NMR (400 MHz, CDCl_3_): δ 8.55 (br, 1H), 7.44 (d, *J* = 4.0 Hz, 1H), 7.33 (d, *J* = 8.0 Hz, 1H), 7.18 (t, *J* = 8.0 Hz, 1H), 7.11 (t, *J* = 8.0 Hz, 1H), 6.83 (d, *J* = 4.0 Hz, 1H), 6.45 (d, *J* = 4.0 Hz, 1H), 6.40 (t, *J* = 4.0 Hz, 1H), 5.48 (d, *J* = 12.0 Hz, 1H), 4.28 (m, 2H), 3.73 (m, 2H), 3.41 (m, 2H), 2.95 (td, *J* = 8.0 Hz, 4.0 Hz, 1H), 1.75 (m, 1H), 1.35 (m, 1H), 0.75 (m, 1H), −0.13 (m, 1H). ^13^C NMR (100 MHz, CDCl_3_): δ 166.3, 164.3, 148.8, 142.9, 135.7, 127.4, 123.9, 122.4, 119.7, 119.6, 111.0, 110.7, 110.2, 108.4, 60.2, 59.2, 44.5, 39.4, 28.2, 26.9, 20.6. HRMS (ESI) *m*/*z* calculated for C_21_H_22_N_3_O_3_ [M+H]^+^ 364.1661, found 364. 1655.

**(3*S*,8a*S*)-3-((1*H*-indol-3-yl)methyl)-2-(4-(dimethylamino) benzyl)hexahydropyrrolo[1,2-*a*]pyrazine-1,4-dione**, **1e**: yield, 61.5%; white solid; mp 195–197°C. ^1^H NMR (400 MHz, CDCl_3_): δ 8.88 (s, 1H), 7.65 (d, *J* = 7.9 Hz, 1H), 7.39–7.31 (m, 1H), 7.24 (d, *J* = 8.6 Hz, 2H), 7.18 (tt, *J* = 8.1, 1.5 Hz, 1H), 7.10 (ddt, *J* = 8.4, 7.1, 1.4 Hz, 1H), 6.96 (s, 1H), 6.74 (d, *J* = 8.2 Hz, 2H), 5.68 (d, *J* = 14.4 Hz, 1H), 4.23 (s, 1H), 3.97 (d, *J* = 14.4 Hz, 1H), 3.83–3.72 (m, 1H), 3.64 (dd, *J* = 14.8, 2.5 Hz, 1H), 3.48–3.29 (m, 2H), 2.98 (d, *J* = 0.9 Hz, 6H), 2.93 (td, *J* = 11.0, 4.7 Hz, 1H), 1.81–1.67 (m, 1H), 1.41–1.25 (m, 1H), 0.79–0.59 (m, 1H), −0.21 (td, *J* = 10.9, 9.7, 2.6 Hz, 1H). ^13^C NMR (100 MHz, CDCl_3_): δ 166.2, 164.7, 135.7, 129.9, 127.4, 124.0, 122.3, 119.7, 119.6, 112.9, 110.0, 108.4, 59.3, 59.0, 45.6, 44.5, 40.8, 28.3, 26.7, 20.6. HRMS (ESI) *m*/*z* calculated for C_25_H_29_N_4_O_2_ [M+H]^+^ 417.2291, found 417.2284.

Characterization data: ^1^H NMR, ^13^C NMR spectra for products **1a–e** (**Pages S3–S13**) and HRMS of **1b, d, e** (**Pages S24–S26**) are shown in the Supplementary Material. General procedure for synthesis of methyl *L*-tryptophanate hydrochloride, 7 and *N*-substituted *L*-tryptophan methyl ester **8a–8e** are presented in the Supplementary Material (**Page S2**). Characterization data: ^1^H NMR, ^13^C NMR spectra for intermediate products 7 (**Pages S14, S15**) and **8a–8e** (**Pages S16–S23**) are shown in the Supplementary Material.

**Scheme 2:**
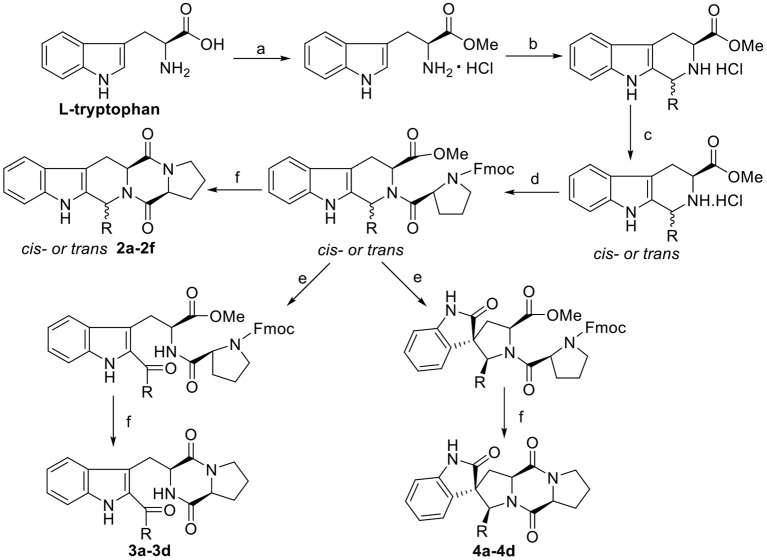
Synthesis route of the target compounds **2a−2f, 3a−3d**, and **4a−4d**. Reaction conditions: (a) SOCl_2_, CH_3_OH; (b) *i*-PrOH, R-CHO, reflux; (c) CH_3_NO_2_/toluene, CIAT; (d) Fmoc-*L*-Pro-Cl, TEA, DCM/Na_2_CO_3_(aq); (e) NBS, AcOH, THF-H_2_O (1:1); (f) morpholine, DCM.

**Scheme 3:**
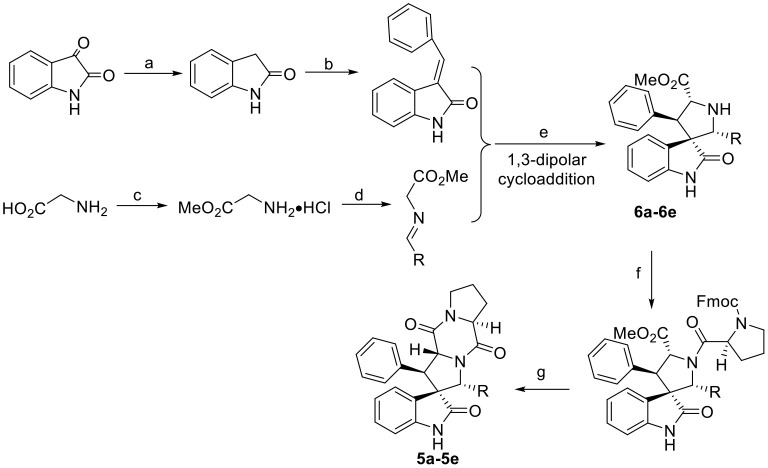
Synthesis route of the target compounds **5a−5e** and intermediate **6a−6e**. Reaction conditions: (a) EtOH, NH_2_NH_2_·H_2_O, NaOH; (b) benzaldehyde, EtOH, piperidine; (c) SOCl_2_, MeOH; (d) aromatic aldehyde, TEA; (e) (*S*)-TF-BiphamPhos/AgAcO, TEA, DCM; (f) Fmoc-*L*-Pro-Cl, TEA, DCM/Na_2_CO_3_(aq); (g) morpholine, DCM.

The synthetic routes of indole DKPs **2a−2f**, **3a−3d**, **4a−4d**, and **5a−5e** are outlined in Scheme 2 and 3. General procedure for the synthesis of these compounds have been described in our previous research (Ma et al., 2013, 2014, 2017).

### Antibacterial and Antifungal Activity

*In vitro* antibacterial and antifungal activities were determined by their minimum inhibitory concentration (MIC) values with a minor modification. The MIC is the lowest concentration of a sample that inhibits the visible growth of a microorganism (Wiegand et al., 2008). Antimicrobial activity of indole DKPs against plant pathogenic fungi (*Colletotrichum gloeosporioides, Valsa mali, Alternaria alternate*, and *Alternaria brassicae*), gram-positive bacteria (*Staphylococcus aureus* and *Bacillus subtilis*), and gram-negative bacteria (*Pseudomonas aeruginosa, Escherichia coli*). The test compounds were completely dissolved in dimethyl sulfoxide (DMSO) and added to a 96-well plate by double dilution of each compound over the range of 0.39–50 μg/mL. Then, potato-glucose liquid medium and a bacteria or fungi suspension were added to each well. The concentration of each microorganism was adjusted to an estimated cell density of ~10^6^ cfu/mL. The plates were kept in an incubator for 24 h at 37°C for bacteria and 72 h at 30°C for fungi. Each experiment was carried out in triplicate. The MIC was determined as the lowest concentration of the first clear well by macroscopic observation under a black background. Positive control samples were penicillin sodium for gram-positive bacteria, streptomycin sulfate for gram-negative bacteria and carbendazim for plant pathogenic fungi. DMSO was used as the blank control.

### Molecular Modeling

The structure of FabH (PDB 1HNJ) was obtained from the RCSB protein Data Bank (http://www.pdb.org/pdb/home/home.do). AutoDock 4.2 was employed to analyze the interactions of the active compounds to the enzyme. All the heteroatoms were removed from the 1HNJ.pdb, to make complex receptor free of any ligand before docking. Water molecule of enzyme were removed, and hydrogen atoms were added in the standard geometry before docking by AutoDock tools. The ligand file was submitted to Chem3D Ultra Visualizing program to minimize to the lowest energy and obtain standard 3D structure in (.pdb) format. Docking runs were carried out using a radius of 48 Å, with coordinates x = 26.819, y = 19.041, z = 28.216, and a spacing of 0.375. Docking was conducted by Lamarckian Genetic Algorithm (LGA). PyMOL (version 0.99; DeLano Scientific, San Carlos, CA, USA) and ligplus v1.4.5 were used to view the graphic.

## Results and Discussion

### Synthesis of Indole DKPs

The general synthetic route for indole DKPs **1a−1e**, **2a−2f**, **3a−3d**, **4a−4d**, and **5a−5e** is outlined in Schemes 1–3. At the outset, a series of fused pentacyclic indole DKPs (**2a−2f**) consisting of a single isomer were synthesized from methyl L-tryptophanate hydrochloride **7** prepared through the esterification and acidification of L-tryptophan. Based on the Pictet-Spengler reaction (reaction condition b, Scheme 2), hydrochloride **7** reacted with six aldehydes to give rise to the corresponding chiral cyclic intermediate. Crystallization-induced asymmetric transformation (CIAT) (reaction condition c, Scheme 2) allowed the stereoselective formation of the *cis*- or *trans* isomer of the chiral intermediate. Then, the intermediates were reacted with Fmoc-L-Pro-Cl, followed by treatment with morpholine as the base to obtain the cyclic peptides by the Schotten-Baumann reaction (reaction conditions e and f, Scheme 2) (Ma et al., 2013). Thus, the target compounds were obtained. In our following study, this method was further extended to synthesize the spiro-pentacyclic indole DKPs (**4a−4d**) (Scheme 2). *N*-bromosuccinimide (NBS) acted as the key reagent for the spiro-rearrangement. Unfortunately, the analog containing an aromatic group was not synthesized successfully. When aromatic aldehydes were employed in the Pictet-Spengler reaction, a series of open-cyclic compounds (**3a−3d**) bearing substitutions at the indole 2-position were obtained, Scheme 2 (Ma et al., 2014). The reason for this failure may be because there were two paths for the reaction with NBS according to the effects between the conjunctive and electrophile. Notably, when the substitution was an aromatic group, the corresponding spiro-pentacyclic indole DKPs failed to form through the NBS oxidative rearrangement. In consideration of this failure, we turned our attention to developing another approach for the preparation of the spiro-pentacyclic scaffold via a 1,3-dipolar cycloaddition of *N*-unprotected 2-oxoindolin-3-ylidenes and azomethine ylides, followed by the above synthetic routes. Finally, spiro-pentacyclic compounds **5a−5e** were obtained successfully, as shown in Scheme 3 (Ma et al., 2017).

**Figure 2:**
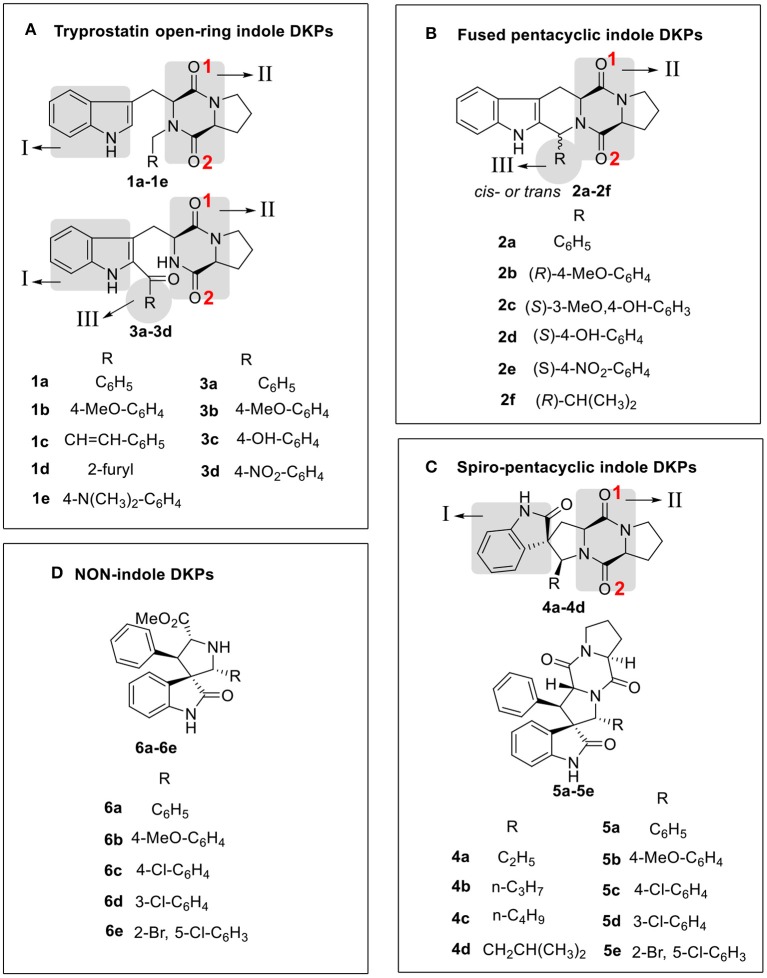
Compound structure of three analogs of indole DKPs and indole analogs **6a−6e**, which were grouped into four framework categories **(A–D)**. These “I,” “II,” “III” represent three subunits of chemical structure of indole DKPs and “1,” “2” represent the position of Oxygen atom on the diketopiperazine ring.

With the three indole DKPs analogs in hand, we focused on the substituted groups. In order to investigate whether the substituted groups attached to the indole or diketopiperazine influence the activity, a series of open-cyclic analogs **1a−1e** with substitution at the diketopiperazine moiety were synthesized (**Scheme 1**). As shown in Figure 2, the obvious difference between **1a−1e** and **3a−3d** are their substituent positions. The substituents of **1a−1e** are at C-2 position of the indole nucleus, while **3a−3d** are *N-*substituted diketopiperazines. In the approach of **1a−1e**, the key intermediates, *N-*substituted *L*-tryptophan methyl esters **8a−8e**, were synthesized efficiently via a one-pot procedure, which included removal of the hydrochloride, condensation and reduction. This synthetic method yielded tryptophan analogs in 54.9% yield, which efficiently shortened the overall reaction time and increased the yield of the indole DKPs. Then, **8a−8e** were treated with Fmoc-L-Pro-Cl and morpholine. Subsequently, we obtained five open-ring indole DKPs, **1a−1e** (Scheme 1). It is worth mentioning that compounds **1b**, **1d**, and **1e** have not been previously reported. In conclusion, the preferred synthetic methods for indole DKPs have been discovered, which delivered 24 indole DKPs (Figure 2).

### Antimicrobial Assay

**Figure 3:**
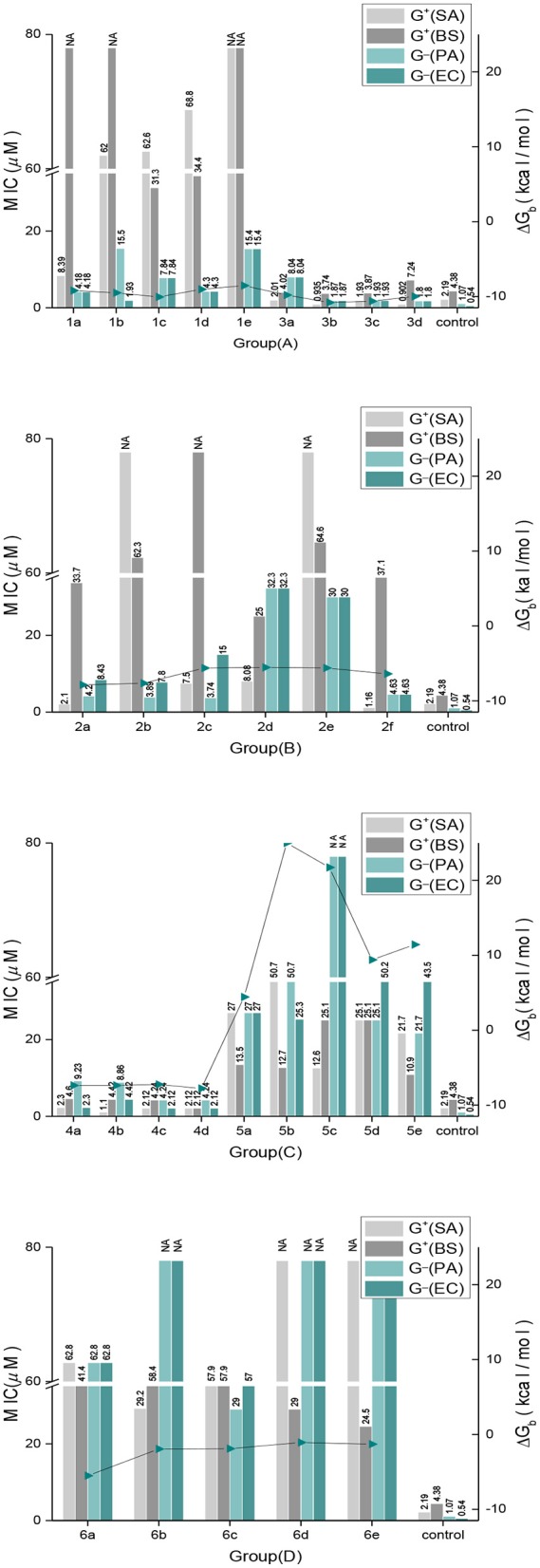
The correlation between antimicrobial activities and the binding free energies.

The antimicrobial activities of the 24 indole DKPs and 5 indole analogs were evaluated against gram-positive bacteria, gram-negative bacteria and 4 plant pathogenic fungi by determining their MICs. The results are listed in Table 1 and Figure 3. Most indole DKPs exhibited good antibacterial activity (1.0–30.0 μM) but moderate antifungal activity (30.0–68.8 μM). For indole analogs **6a−6e**, their antimicrobial activity remarkably decreased or disappeared (most of them >57.9 μM). The best antibacterial effects against the tested strains were found for indole DKPs **3a−3d** and **4a−4d**, which showed MIC values in range of 0.90–9.23 μM. For compounds **1a−1e** and **2a−2f**, except **1a**, **2a**, **2c**, and **2f**, most of them showed modest (30.0–68.8 μM) or no antibacterial activity against the tested bacteria. However, most of the remaining compounds **5a−5e** and **6a−6e** had little or no bioactivity against any strain (most of them >50.7 μM). In view of these results, all tested compounds were divided into three groups according to their antibacterial activities: high activity (**3a−3d** and **4a−4d**), medium activity (**1a−1e** and **2a−2f**), and low activity (**5a−5e** and **6a−6e**). It is noteworthy that some of the compounds were highly active against the tested bacteria, and the compounds showed activity higher than that of penicillin sodium. For example, compounds **2a**, **2f**, **3a**, **3b**, **3c**, **3d**, **4b**, **4c**, and **4d** were more active than the positive control of penicillin sodium (2.19 μM) against *S. aureus* at the MICs 2.10, 1.16, 2.01, 0.94, 1.93, 0.90, 1.10, 2.12, and 2.12 μM, respectively. Compounds **3a**, **3b**, **3c**, **4a**, **4b**, **4c**, and **4d** were higher or comparable to the positive control of penicillin sodium (4.38 μM) against *B. subtilis* at the MICs 4.02, 3.74, 3.87, 4.60, 4.42, 4.42, and 2.12 μM, respectively. In addition, compounds **1b**, **3b**, **3c**, and **3d** had good activity against *E. coli* at the MICs 1.98, 1.87, 1.93, and 1.80 μM, respectively, in comparison with positive control of streptomycin sulfate (0.54 μM). However, only **4a−4d** displayed broad-spectrum antimicrobial activity against the tested bacteria and fungi with MIC values ranging from 1.10 to 73.7 μM. Fortunately, **4b** was found to show moderate antifungal activity range (17.7–35.4 μM) against the four test fungi, and similarly, **4a** (18.4–36.9 μM) also had moderate antifungal activity against the three test fungi except for *V. mali*. Moreover, **4c** had moderate antifungal activity with MIC of 34.0 μM against *A, alternate, V. mali* and *A. brassicae*. Besides that, **1c** was found to have moderate antifungal activity with MIC of 31.3 μM against *C. gloeosporioides* and *V. mali*. **3b** and **3c** were found to have moderate antifungal activity against *V. mali* with MIC values of 30.0 and 31.0 μM, respectively. Furthermore, fumitremorgin B, verruculogen and cyclotryprostatin A, which we previously obtained from the endophytic fungus *Aspergillus tamarii* in *Ficus carica* L, showed good anti-phytopathogenic activity against *Fusarium graminearum, Phytophthora capsici*, and *V. mali* (MICs: 12.5–25 μg /mL) compared to the positive control Nystatin (MICs: 6.25–12.5 μg /mL) (Zhang, 2012). Therefore, the antifungal activity of indole DKPs deserves further study.

**Table 1:** Antimicrobial Activity of the Four Structure Groups against tested bacteria and plant pathogenic fungi.

**Structure group**	**Compound**	**Minimum inhibitory concentrations [MICs**, *****μ*******g/mL (*******μ*******M)]**								**Clog *P*^a^**
		**Antibacterial activity**				**Antifungal activity**				
		**Gram-positive**		**Gram-negative**						
		**SA**	**BS**	**PA**	**EC**	**CG**	**VM**	**AA**	**AB**	
**A**	**1a**	3.13 (8.39)	NA^b^	1.56 (4.18)	1.56 (4.18)	NA	25 (67.0)	25 (67.0)	25 (67.0)	3.3254
	**1b**	25 (62.0)	NA	6.25 (15.5)	0.78 (1.93)	25 (62.0)	25 (62.0)	50 (124)	NA	3.2444
	**1c**	25 (62.6)	12.5 (31.3)	3.13 (7.84)	3.13 (7.84)	12.5 (31.3)	12.5 (31.3)	25 (62.6)	25 (62.6)	4.0254
	**1d**	25 (68.8)	12.5 (34.4)	6.25 (17.2)	1.56 (4.30)	25 (68.8)	25 (68.8)	25 (68.8)	25 (68.8)	2.5014
	**1e**	NA	NA	3.13 (7.52)	6.25 (15.4)	50 (120)	25 (60.1)	25 (60.1)	NA	3.4904
	**3a**	0.78 (2.01)	1.56 (4.02)	3.13 (8.04)	3.13 (8.04)	NA	25 (64.6)	NA	25 (64.6)	2.5510
	**3b**	0.39 (0.94)	1.56 (3.74)	1.56 (3.74)	0.78 (1.87)	25 (59.9)	12.5 (30.0)	25 (59.9)	25 (59.9)	2.5924
	**3c**	0.78 (1.93)	1.56 (3.87)	1.56 (3.87)	0.78 (1.93)	25 (62.0)	12.5 (31.0)	25 (62.0)	25 (62.0)	2.1390
	**3d**	0.39 (0.90)	3.13 (7.24)	1.56 (3.62)	0.78 (1.80)	NA	25 (57.9)	25 (57.9)	NA	2.4560
**B**	**2a**	0.78 (2.10)	12.5 (33.7)	1.56 (4.20)	3.13 (8.43)	50 (135)	NA	NA	50 (135)	2.8014
	**2b**	NA	25 (62.3)	1.56 (3.89)	3.13 (7.80)	NA	25 (62.3)	NA	NA	2.7204
	**2c**	3.13 (7.50)	NA	1.56 (3.74)	6.25 (15.0)	25 (60.0)	NA	25 (60.0)	25 (60.0)	1.9836
	**2d**	3.13 (8.08)	25 (64.6)	12.5 (32.3)	12.5 (32.3)	25 (64.6)	25 (64.6)	25 (64.6)	25 (64.6)	2.1344
	**2e**	NA	NA	12.5 (30.0)	12.5 (30.0)	25 (60.1)	25 (60.1)	25 (60.1)	25 (60.1)	2.5444
	**2f**	0.39 (1.16)	12.5 (37.1)	1.56 (4.63)	1.56 (4.63)	25 (74.1)	25 (74.1)	NA	25 (74.1)	2.6904
**C**	**4a**	0.78 (2.30)	1.56 (4.60)	3.13 (9.23)	0.78 (2.30)	12.5 (36.9)	25 (73.7)	6.25 (18.4)	12.5 (36.9)	1.1819
	**4b**	0.39 (1.10)	1.56 (4.42)	3.13 (8.86)	1.56 (4.42)	12.5 (35.4)	12.5 (35.4)	6.25 (17.7)	12.5 (35.4)	1.7109
	**4c**	0.78 (2.12)	1.56 (4.24)	1.56 (4.24)	0.78 (2.12)	25 (68.1)	12.5 (34.0)	12.5 (34.0)	12.5 (34.0)	2.2399
	**4d**	0.78 (2.12)	0.78 (2.12)	1.56 (4.24)	0.78 (2.12)	12.5 (34.0)	25 (68.1)	25 (68.1)	25 (68.1)	2.1099
	**5a**	12.5 (27.0)	6.25 (13.5)	12.5 (27.0)	12.5 (27.0)	25 (54.0)	25 (54.0)	25 (54.0)	25 (54.0)	3.3239
	**5b**	25 (50.7)	6.25 (12.7)	25 (50.7)	12.5 (25.3)	25 (50.7)	25 (50.7)	25 (50.7)	25 (50.7)	3.2429
	**5c**	6.25 (12.6)	12.5 (25.1)	NA	NA	50 (101)	50 (101)	50 (101)	50 (101)	4.0369
	**5d**	12.5 (25.1)	12.5 (25.1)	12.5 (25.1)	25 (50.2)	50 (101)	50 (101)	50 (101)	50 (101)	4.0369
	**5e**	12.5 (21.7)	6.25 (10.9)	12.5 (21.7)	25 (43.5)	50 (86.9)	50 (86.9)	25 (43.5)	50 (86.9)	4.8999
**D**	**6a**	25 (62.8)	12.5 (41.4)	25 (62.8)	25 (62.8)	NA	50 (126)	NA	50 (126)	3.2219
	**6b**	12.5 (29.2)	25 (58.4)	NA	NA	50 (117)	50 (117)	50 (117)	25 (29.2)	3.1409
	**6c**	25 (57.9)	25 (57.9)	12.5 (29.0)	25 (57.9)	25 (57.9)	25 (57.9)	25 (57.9)	25 (57.9)	3.9349
	**6d**	NA	12.5 (29.0)	NA	NA	50 (116)	50 (116)	NA	50 (116)	3.9349
	**6e**	NA	12.5 (24.5)	NA	NA	50 (98.0)	NA	25 (49.0)	50 (98.0)	4.7979
	Streptomycin sulfate	—^c^	—	1.56 (1.07)	0.78 (0.54)	—	—	—	—	
	Penicillin sodium	0.78 (2.19)	1.56 (4.38)	—	—	—	—	—	—	
	Ketoconazole	—	—	—	—	12.5 (23.5)	12.5 (23.5)	6.25 (11.8)	12.5 (23.5)	

*Gram positive bacteria: SA, Staphylococcus aureus; BS, Bacillus subtilis; Gram negative bacteria: PA, Pseudomonas aeruginosa; EC, Escherichia coli; Fungi: CG, colletotrichum gloeosporioides; VM, valsa mali; AA, alternaria alternate; AB, alternaria brassicae*.

a *Theoretical estimated using ChemBioDraw Ultra 14.0 program*.

b *NA, not active in assay (MIC > 50 μg/mL)*.

c *Not determined*.

### Lipophilicity

Lipophilicity is an important factor for exploring the mechanism of action of antimicrobial agent against pathogenic bacteria (Shi et al., 2018). Therefore, the theoretical partition coefficients (Clog *P*) were considered for the current discussion. The antimicrobial activity of indole DKPs was also found to have a significant relationship with their lipophilicity. We found that many of the synthesized molecules have encouraging antimicrobial activities, as well as lower Clog *P* values, such as high activity compounds **3a−3d** and **4a−4d** (Clog *P*, 1.18–2.59), medium activity compounds **1a−1e** and **2a−2f** (Clog *P*, 1.98–4.03). Moreover, low activity **5a−5e** and **6a−6e** had higher Clog *P* values (3.14–4.80). Shi et al. have reported that ferulic acid and ferulic acid alkyl esters (FAC2) with 1.4212 and 2.1762 of Clog *P* values showed higher antibacterial activity against *E. coli* as compared with other similar compounds having higher Clog *P* values (Shi et al., 2018). Moreover, this similar trend also was reported by Wu et al. They found auranofin analogs with lower log *P* values (1–2) exhibited better activity than some analogs with higher log *P* values (>3) against *S. aureus, E. coli*, and *P. aeruginosa* (Wu et al., 2019). In view of the results, we can suggest that high hydrophobicity promotes reagents uptake by bacteria because of the lower Clog *P* value. Thus Clog *P* values between 2 and 3 are often considered optimal for oral drugs (Blaser et al., 2012). It has been proposed that ligands with a Clog *P* < 5 have a more promising druglikeness profile (Schübler et al., 2017). In general, all the indole DKPs we synthesized endowed with moderate Clog *P* values (1.18–4.90) that indicated the compounds can be as drug candidates.

### Structure-Activity Relationship

The structure-activity relationship (SAR) of indole DKPs can be established from the results of the antimicrobial activities reported in Table 1. Analysis of the SAR of the indole DKPs provides the link between the structure and the activity, which offers clues for structural modifications that can improve the activity. SAR analysis is important in understanding the mechanisms of antibacterial activity for indole DKPs. First, the skeleton of indole DKPs, which is comprised of two cores, an indole and a diketopiperazine, was shown to be important for antimicrobial activity. Indole analogs **6a−6e** were less efficient as antibacterial agents, while most indole DKPs exhibited good activity. Second, a change in the skeleton greatly affected the antibacterial activity. We can see that there is a tendency for decreasing influence in the following order: tryprostatin open-ring > fused pentacyclic > spiro-pentacyclic indole DKPs. For most of tested bacteria, fused pentacyclics **2a−2f** were more active than the spiro-pentacyclics **5a−5e** but **2a−2f** were weaker inhibitors than the tryprostatin open-ring compounds **3a−3d**. However, spiro-pentacyclic compounds **4a−4d** showed better activities than the open-ring compounds **1a−1e** and fused pentacyclics **2a−2f**. In addition, tryprostatin open-ring compounds **3a−3d** were more active than **1a−1e**. Third, the kind of substituent and its position also significantly affected the antimicrobial activity. For the spiro-pentacyclic indole DKPs, alkyl substituents improved the antibacterial activity, while aromatic substituents decreased the antibacterial activity. For tryprostatin open-ring indole DKPs, substituents at C-2 position of the indole nucleus improved the antibacterial activity, while *N-*substituted diketopiperazines decreased the antibacterial activity. These results can provide some useful information for future work.

### Molecular Docking

The FabH active site generally contains a catalytic triad tunnel consisting of Cys112, His244, and Asn274. Affecting these amino acid resides may greatly influence, inhibit or even stop the enzyme's catalytic activity (Davies et al., 2000). The direct result of this would be that fatty acid biosynthesis cannot proceed smoothly, the energy supply to the organism would not be sufficient, the components of all cell membranes could not be formed, and antimicrobial activity would be displayed (Cheng et al., 2013). We therefore carried out molecular docking studies of the indole DKPs with the crystal structure of *E. coli* FabH (entry 1HNJ in the Protein Data Bank) to reveal the indole DKPs binding mode. The molecular docking study can provide useful information for the active mechanism of indole DKPs. The docking results are shown in Table 2 and Figure 3.

**Table 2:** The binding affinity of three group indole DKPs with *E. coli* FabH.

**Structure group**	**Compound**	**Δ*G*_b_^a^ (kcal/mol)**	**Hydrogen bond^a^**	**Hydrophobic residue^b^**	**π−π interaction^b^**
**A**	**1a**	−9.20	N_I_−*H*···O/Gly209	Cys112, Asn247, Ile250, Ala216, Leu220, His244, Ala246, Phe304	–*^c^*
	**1b**	−9.50	N_I_−*H*···O/Gly209 O_II−1_···H–N/Asn274	Cys112, Leu220, Ile250, Ala246, His244, Asn247, Ile156, Phe304	–
	**1c**	−10.59	N_I_−*H*···O/Gly209 O_II−1_···H–N/Asn274	Cys112, Leu220, Ile250, Ala246, His244, Asn247, Ile156, Phe304	–
	**1d**	−9.03	N_I_−*H*···O/Gly209 O_II−1_···H–N/Asn274	Cys112, Ala246, Ile250, Asn247, Ile156	–
	**1e**	−8.54	N_I_−*H*···O/Gly209	Cys112, Asn247, Val212, Ala216, Ile250, Ile156, His244, Leu220, Ala246, Phe304	–
	**3a**	−9.79	O_II−2_···H–N/Asn247 N_II−3_−*H*···O/Ala246	Ile250, His244, Leu189, Leu220, Val212, Cys112	–
	**3b**	−10.79	O_II−2_···H–N/Asn247 N_I_ -H···O/Phe304	Ile250, Ala246, Leu220, Met207, Cys112, Leu189	–
	**3c**	−10.61	O_II−2_···H–N/Asn247 N_I_−*H*···O/Phe304 O_III−OH_−*H*···O/His244	Ala246, His244, Leu220, Ile250, Cys112, Met207	–
	**3d**	−9.99	O_II−2_···H–N/Asn247 N_I_−*H*···O/Phe304 O _III−NO2_···H–N/His244	Ala246, Ile250, Leu220, Met207, Phe157, Cys112, Leu189	–
**B**	**2a**	−7.87	O_II−2_···H–N/Asn247	Ile156, Met207, Ile155, Phe213, Asn210	Ph_I_···Ph/Phe213
	**2b**	−7.65	O_III−MeO_···H–N/Phe304	–*^c^*	–
	**2c**	−5.63	O_III−OH_−*H*···O/Gly152	Met207, Ile156, Ala246, Gly209, Phe213, Asn210	Ph_I_···Ph/Phe213
	**2d**	−5.45	O_III−OH_−*H*···O/Gly152	Met207, Ala246, Asn210, Phe213, Gly209	Ph_I_···Ph/Phe213
	**2e**	−5.61	–	Met207, Ala246, Asn210, Phe213, Gly209	Ph_I_···Ph/Phe213
	**2f**	−6.40	O_II−1_···H–N/Asn274	Cys112, Phe213, Met207	–
**C**	**4a**	−7.37	O_II−2_···H–N/Asn247	Ile156, Ala246, Val212	–
	**4b**	−7.37	O_II−2_···H–N/Asn247	Ile156, Ala246, Val212	–
	**4c**	−7.24	O_II−2_···H–N/Asn247	Phe213, Ile156, Ala246, Val212	–
	**4d**	−7.78	O_II−2_···H–N/Asn247 O_I_ ···H–N/Asn274	Ile156, Ala246, Val212	–

a *ΔG_b_ and hydrogen bond were obtained from AutoDock 4.2*.

b *Hydrophobic residues and π−π interaction were obtained from https://proteins.plus/*.

c *No interactions found by AutoDock 4.2 and the PoseView interaction model*.

*In silico* studies revealed that most of the synthesized molecules showed good binding free energy (ΔG_b_, kcal/mol) toward the target protein ranging from −5.45 to −10.79 kcal/mol (Table 2). Moreover, the changes in ΔG_b_ coincided well with the MIC values obtained for most of the compounds, as shown in Figure 3. Specifically, compounds **3a−3d** with good activity exhibited a very low ΔG_b_, from −10.79 to −9.79 kcal/mol. Compounds **2a−2f** with moderate activity exhibited ΔG_b_ values from −7.87 to −5.45 kcal/mol. Moreover, compounds **5a−5e** and **6a−6e** had the lowest bioactivities and also revealed poor binding free energy values compared to the other indole DKPs, their binding free energies were 4.43, 25.00, 21.73, 9.41, 11.43 kcal/mol and −5.51, −1.94, −1.90, −1.07, −1.29 kcal/mol, respectively (Figure 3). Furthermore, no hydrogen bonds and little hydrophobic contacts could be found in the best binding conformations for **5a−5e** and **6a−6e**. With the observations from the biological assay data and the molecular docking results, might suggest that the antibacterial activities of these compounds are seemingly derived from the interaction between the compounds and the enzyme FabH. However, there was not always a correlation between the *in vitro* activity and *in silico* study results. Although compounds **1a−1e** showed good binding affinity for FabH with ΔG_b_ values between −10.59 and −8.54 kcal/mol, they showed low antimicrobial activity against *S. aureus* and *B. subtilis*, but good activity against *P. aeruginosa* and *E. coli*, as shown in Figure 3. The results suggest that there may be an alternate target in addition to FabH for compounds **1a−1e**.

In addition, the binding affinity was also evaluated by hydrogen bonding, hydrophobic interactions and π−π interactions. As shown in Table 2, hydrogen bonding and hydrophobic interactions are important interactions between the compounds and FabH. For example, Analogs **1b−1d** exhibited hydrogen bonds via their N_I_H (amino group of the indole, Figure 2) with the carbonyl oxygen of Gly209, and their O_II−1_ (the backbone oxygen atom of proline, Figure 2) with the side chain NH from Asn274. Analogs **3b−3d** exhibited hydrogen bonds via their O_II−2_ (the oxygen atom of the *N-*acetyl group, Figure 2) with the side chain NH from Asn247, and their N_I_H with the backbone carbonyl oxygen of Phe304. There is also a hydrogen bond between their substituent (in subunit III, Figure 2) and His244. Analogs **2a−2d** and **2f** formed hydrogen bonds with Asn247, Phe304, Gly152, or Asn274. Furthermore, there is a π−π interaction between the phenyl ring of compound **2a** and the benzyl ring of Phe213, and this interaction is also observed for **2c, 2d**, and **2e** (Table 2), which further stabilized the interaction of these compounds with the enzyme. Analogs **4a−4d** exhibited hydrogen bonds via their O_II−2_ with the side chain NH of Asn247. Compound **4d** also formed a hydrogen bond via its O_I_ (carbonyl oxygen of indole moiety, Figure 2) with the side chain NH of Asn274. In general, two subunits (subunit I and subunit II, Figure 2) of the indole DKPs and a substituent group (subunit III, Figure 2) of the indole DKPs could form hydrogen bonds with FabH. In addition, hydrophobic interactions were observed between the indole DKPs and a set of amino acids in the catalytic center of *E. coli* FabH (Table 2).

**Figure 4:**
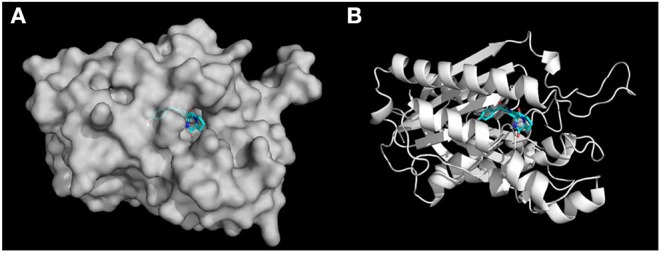
**(A)** Docking simulation of binding mode between compound **3c** and FabH. The receptor FabH is shown in surface view, while the structure of compound **3c** is shown as sticks. **(B)** The 3D diagram of compound **3c** interaction with the FabH active site. The proteins are shown in cartoon representation with the ligands drawn as sticks.

**Figure 5:**
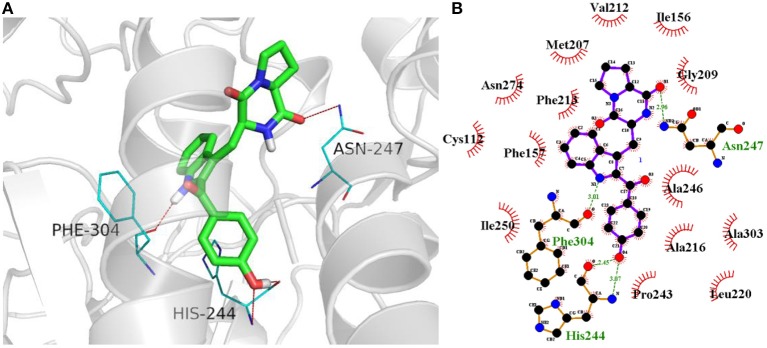
**(A)** Docking of compound **3c** (colored in green) into the active site of FabH (gray, PDB: 1HNJ) via hydrogen bond with Asn247, Phe304, and His244(colored in blue). Likely hydrogen bonds are indicated by red dashed lines (four hydrogen bonds). **(B)** The details of interactions were displayed by ligplus v1.4.5.

In terms of the highly active analogs **3a−3d**, specifically compound **3c**, as expected from the docking calculations of the indole DKPs, showed significant binding affinity to FabH. Compounds **3c** forms three hydrogen bonds with the key residues His244, Asn247, and Phe304, and fits best in the FabH active pocket with a very low interaction energy. Furthermore, **3c** reveals an intriguing binding conformation in this pocket. As Figure 4A shows, the FabH binding site buries the indole ring of **3c** in its cavity while leaving the DKP ring floating in the hydrophobic pocket. The backbone and chains of **3c** were nicely nestled (Figure 4B). Additionally, compound **3d** shows a similar binding mode to that of **3c**. Figure 5 show detailed illustrations of interactions between compound **3c** and FabH. First, **3c** fills a hydrophobic region at the top of the active site tunnel and exhibits hydrogen bonds via its *N-*acetyl group O_II−2_ with the side chain NH from Asn247. Second, its indole NH is linked by a hydrogen bond to the backbone carbonyl oxygen of Phe304 on one side of the tunnel. Third, **3c** exhibits hydrogen bonds via its hydroxyl groups with the backbone NH and O of the catalytic residue His244 and fills the bottom of the active tunnel. Hydrophobic interactions between **3c** and FabH appear to be additional important factors in the binding mode, which are made up of Ala246, Leu220, Ile250, Cys112, and Met207 and which enhance the binding affinity. All of these results may explain why **3c** had good antibacterial activity.

## Conclusion

In conclusion, a series of indole DKPs were designed and synthesized. Most of the analogs exhibited good antibacterial activity against the four tested bacteria and showed moderate antifungal activity against the four tested fungal species. Some of the compounds displayed lower MIC values than the positive control on some of the tested bacteria. We carried out the first SAR investigation into the antimicrobial activity of indole DKPs. The SAR results showed that the indole DKPs skeleton has significant effects on the activity. All of the results revealed that the compounds are potential antibacterial agents, which could be further optimized and developed as new antibacterial agents. Furthermore, based on the biological assay data and molecular docking, there could be some correlation between the bioactivity and the FabH inhibitory activity of these compounds. The docking results further suggested that **3c** is a potential FabH inhibitor. However, further experimental studies are needed to confirm this hypothesis, which is our next research focus.

## Data Availability Statement

All datasets generated for this study are included in the article/Supplementary Material.

## Author Contributions

BJ and YM conceived and designed the experiments. BL performed the experiments. PC performed the molecular docking. YH analyzed the data. BJ wrote the paper. YM interpreted the data and revised the manuscript. RZ revised the manuscript.

### Conflict of Interest

The authors declare that the research was conducted in the absence of any commercial or financial relationships that could be construed as a potential conflict of interest.
