# Supplementary material for: Synthesis, Antimicrobial Activity, Structure-Activity Relationship, and Molecular Docking Studies of Indole Diketopiperazine Alkaloids
Source: Front Chem. 2019 Nov 29;7:837. doi: 10.3389/fchem.2019.00837 (PMC6897290; doi:10.3389/fchem.2019.00837)
Supplement: Supplementary file 1 [file Data_sheet_1.docx]

*Supporting information for:*

Synthesis, Antimicrobial Activity, Structure Activity Relationship and Molecular Docking Studies of Indole Diketopiperazine Alkaloids

Bin Jia^1^, Yang-min Ma^1^ ^*^, Bin Liu^2,3^, Pu Chen^1^, Yan Hu^1^ and Rui Zhang^4^

^1^Shaanxi Key Laboratory of Chemical Additives for Industry, College of Chemistry and Chemical Engineering, Shaanxi University of Science and Technology, Xi’an, China

^2^School of Pharmacy, Shaanxi Institute of International Trade & Commerce, Xi’an, China

^3^Collaborative innovation center of green manufacturing technology for traditional Chinese medicine in Shaanxi province, Xi’an, China

^4^School of Arts and Sciences, Shaanxi University of Science and Technology, Xi’an, China

*Correspondence author. E-Mail: [mym63@sina.com](mailto:mym63@sina.com) Tel.: +86-29-86168312 Fax: +86-29-86168312.

**Contents**

General procedure for the synthesis of compounds **7** and **8a**-**8e** Page S2-S3

NMR Spectra and for Synthetic Compounds **1a**-**1e**, **7**, **8a**-**8e** Page S3-S23

HRMS of **1b**, **1d** and **1e** Page S24-S26

**Synthesis of methyl L-tryptophanate hydrochloride, 7.**

To CH_3_OH (50mL) was added dropwise SOCl_2_ (3.27 mL, 45 mmol) at 0 °C, and the mixture was stirred at 0 °C for 30 min and subsequently at room temperature for 2 h, followed by addition of L**-**tryptophan (6.12 g, 30 mmol). The mixture was then refluxed for 5 h and volatile components were removed under reduced pressure to afford the crude product, which was recrystallized in a mixture of ethanol and ethyl ether 5:1 (v/v) to afford hydrochloride **7** (7.09g). yield, 93%; white powder; mp 217~219 °C. ^1^H NMR (400 MHz, DMSO**-**d_6_): δ 11.15 (br, 1H), 8.65**-**8.57 (br, 3H), 7.52 (d, *J* = 8.0 Hz, 1H), 7.39 (d, *J* = 8.0 Hz, 1H), 7.26 (s, 1H), 7.10 (t, *J* = 8.0 Hz, 1H), 7.01 (t, *J* = 8.0 Hz, 1H), 4.24 (m, 1H), 3.65 (s, 3H), 3.30 (m, 2H); ^13^C NMR (100 MHz, DMSO**-**d_6_): δ 170.2, 136.7, 127.4, 125.5, 121.6, 119.1, 118.5, 112.0, 106.8, 53.1, 51.3, 26.5. IR (KBr): 3250, 2961, 1745, 1238 cm^-1^.

**General procedure for synthesis of N-substituted L-tryptophan methyl ester 8a-8e.**

To a solution of methyl L-tryptophanate hydrochloride **7** (2.03 g, 8 mmol) in methanol (20 mL) was added triethylamine (0.80 g, 8 mmol) at 0 °C, and the mixture was stirred at 0 °C for 1 h, followed by corresponding aldehyde in methanol (10 mL) was added dropwise at N_2_ atmosphere. Subsequently, the mixture was stirred at 0 °C for 3 h, and NaBH_4_ (0.36 g, 9.6 mmol) was added to the reaction system through three times. Then the reaction was stirred at 0 °C for 1 h. Water was added and extracted with CH_2_Cl_2_ (3×10 mL). The combined organic phases were dried over MgSO_4_ and concentrated under reduced pressure. The residue was recrystallized in a mixture of chloroform and methanol to afford product amines **8a-8e**.

**methyl benzyl-L-tryptophanate**, **8a**: yield, 73.2%; colorless solid; mp 106-108 °C. ^1^H NMR (400 MHz, CDCl_3_): δ 8.21 (br, 1H), 7.63 (d, *J* = 8.0 Hz, 1H), 7.36 (d, *J* = 8.0 Hz, 1H), 7.33-7.24 (m, 5H), 7.22 (m, 1H), 7.16 (m, 1H), 7.02 (d, *J* = 4.0 Hz, 1H), 3.89 (d, *J* = 12.0 Hz, 1H), 3.74 (t, *J* = 4.0 Hz, 2H), 3.67 (s, 3H), 3.28-3.26 (m, 2H), 1.99 (s, 1H). ^13^C NMR (100 MHz, CDCl_3_): δ 175.4, 139.7, 136.2, 128.4, 128.2(d), 127.5, 127.1(d), 122.9, 122.1, 119.4, 118.8, 111.2, 111.1, 61.2, 52.2, 51.8, 29.3. IR (KBr): 3460, 3150, 2858, 1745, 1611, 1501, 747, 699 cm^-1^.

**methyl (4-methoxybenzyl)-L-tryptophanate**, **8b**: yield, 71.9%; colorless solid; mp 108-110 °C. ^1^H NMR (400 MHz, CDCl_3_): δ 8.24 (br, 1H), 7.61 (d, *J* = 8.0 Hz, 1H), 7.36 (d, *J* = 8.0 Hz, 1H), 7.23 (m, 1H), 7.18 (s, 1H), 7.16 (s, 1H), 7.15 (m, 1H), 7.01 (dt, *J* = 4.0 Hz, 1H), 6.84 (t, *J* = 4.0 Hz, 1H), 6.82 (t, *J* = 4.0 Hz, 1H), 3.81 (s, 3H), 3.78 (s, 1H), 3.72 (t, *J* = 4.0 Hz, 2H), 3.67 (s, 3H), 3.25-3.14 (m, 2H), 1.94 (s, 1H). ^13^C NMR (100 MHz, CDCl_3_): δ 175.4, 158.7, 136.2, 131.8, 129.4(d), 127.5, 122.9, 122.1, 119.4, 118.8, 113.7(d), 111.3, 111.2, 61.1, 55.3, 51.8, 51.6, 29.3. IR (KBr): 3418, 3303, 3017, 2855, 1728, 1603, 1454, 807 cm^-1^.

**methyl cinnamyl-L-tryptophanate**, **8c**: yield, 69.8%; colorless solid; mp 86-88 °C. ^1^H NMR (400 MHz, CDCl_3_): δ 8.15 (br, 1H), 7.66 (d, *J* = 8.0 Hz, 1H), 7.39 (d, *J* = 8.0 Hz, 1H), 7.30-7.25 (m, 5H), 7.23 (m, 1H), 7.17 (m, 1H), 7.10 (d, *J* = 4.0 Hz, 1H), 6.46 (d, *J* = 16.0 Hz, 1H), 6.23 (m, 1H), 3.77 (m, 1H), 3.66 (s, 3H), 3.48 (m, 2H), 3.28-3.15 (m, 2H), 1.85 (s, 1H). ^13^C NMR (100 MHz, CDCl_3_): δ 175.4, 137.0, 136.2, 131.6, 128.5(d), 127.7, 127.5, 127.4, 126.3(d), 123.0, 122.2, 119.6, 118.8, 111.3, 111.2, 61.2, 51.9, 50.2, 29.4. IR (KBr): 3446, 3290, 2840, 1731, 1627, 1504, 1461, 740, 690 cm^-1^.

**methyl (furan-2-ylmethyl)-L-tryptophanate**, **8d**: yield, 66.7%; colorless solid; mp 97-99 °C. ^1^H NMR (400 MHz, CDCl_3_): δ 8.29 (br, 1H), 7.61 (d, *J* = 8.0 Hz, 1H), 7.36 (d, *J* = 8.0 Hz, 1H), 7.31 (m, 1H), 7.23 (m, 1H), 7.16 (m, 1H), 7.01 (d, *J* = 4.0 Hz, 1H), 6.29 (m, 1H), 6.13 (m, 1H), 3.86 (d, J = 16.0 Hz, 1H), 3.76 (m, 2H), 3.65 (s, 3H), 3.26-3.15 (m, 2H), 2.06 (s, 1H). ^13^C NMR (100 MHz, CDCl_3_): δ 175.0, 153.1, 142.0, 136.2, 127.4, 123.0, 122.1, 119.4, 118.7, 111.2, 110.9, 110.1, 107.4, 60.9, 51.9, 44.7, 29.3. IR (KBr): 3460, 3291, 2847, 1747, 1635, 1504, 1471, 1021 cm^-1^.

**methyl (4-(dimethylamino)benzyl)-L-tryptophanate**, **8e**: yield, 77.5%; colorless solid; mp 127-128 °C. ^1^H NMR (400 MHz, CDCl_3_): δ 8.18 (br, 1H), 7.61 (d, *J* = 8.0 Hz, 1H), 7.36 (d, *J* = 8.0 Hz, 1H), 7.22 (m, 1H), 7.15 (m, 1H), 7.13 (m, 2H), 7.02 (d, *J* = 4.0 Hz, 1H), 6.69 (m, 2H), 3.78 (m, 2H), 3.66 (s, 3H), 3.63 (d, *J* = 16.0 Hz, 1H), 3.24-3.14 (m, 2H), 2.95 (s, 6H), 1.90 (s, 1H). ^13^C NMR (100 MHz, CDCl_3_) δ: 175.5, 149.9, 136.2, 129.2, 127.6, 127.5, 122.9, 122.0, 119.4, 118.9, 112.7, 111.3, 111.1, 61.1, 51.7, 51.6, 40.8, 29.3.

NMR Spectra for Synthetic Compounds **1a**-**1e**, **7**, **8a**-**8e**


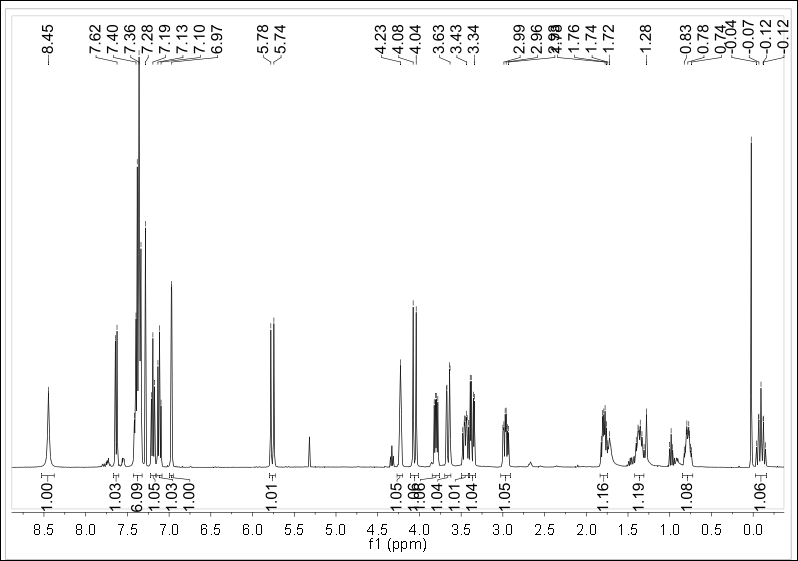

**^1^H-NMR (400 MHz, CDCl_3_) Spectrum of Compound 1a**


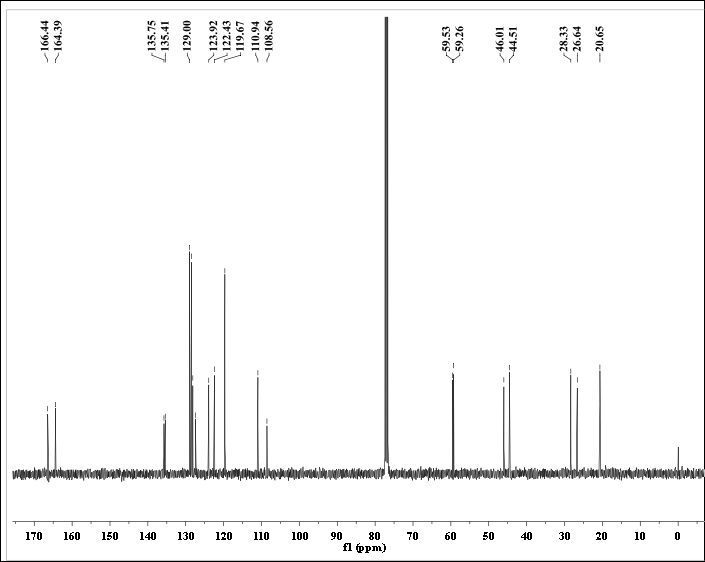

**^13^C-NMR (100 MHz, CDCl_3_) Spectrum of Compound 1a**


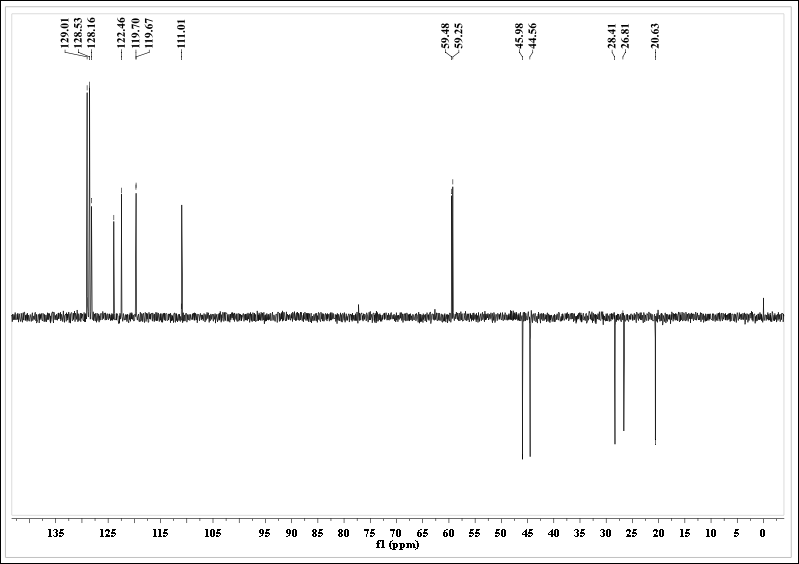


**DEPT-135 Spectrum of Compound 1a**


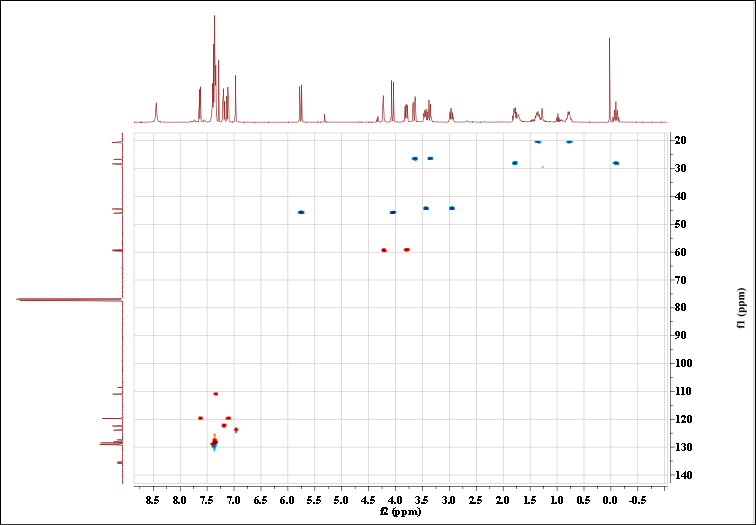


**HSQC of Compound 1a**


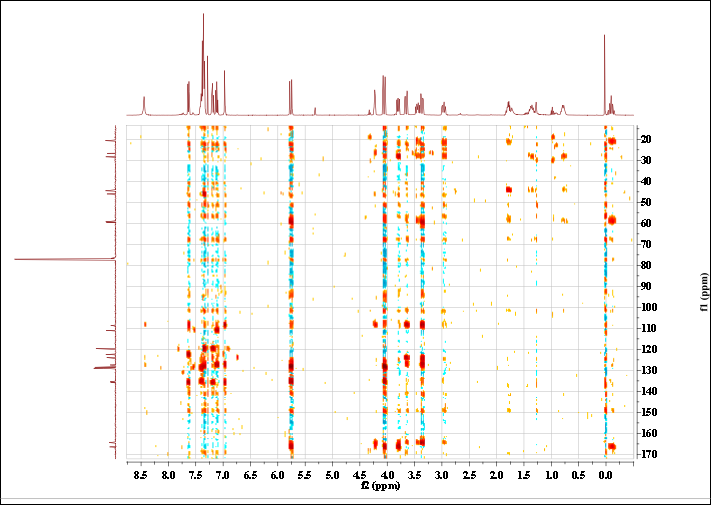


**HMBC of Compound 1a**

**^1^H-NMR (400 MHz, CDCl_3_) Spectrum of Compound 1b**


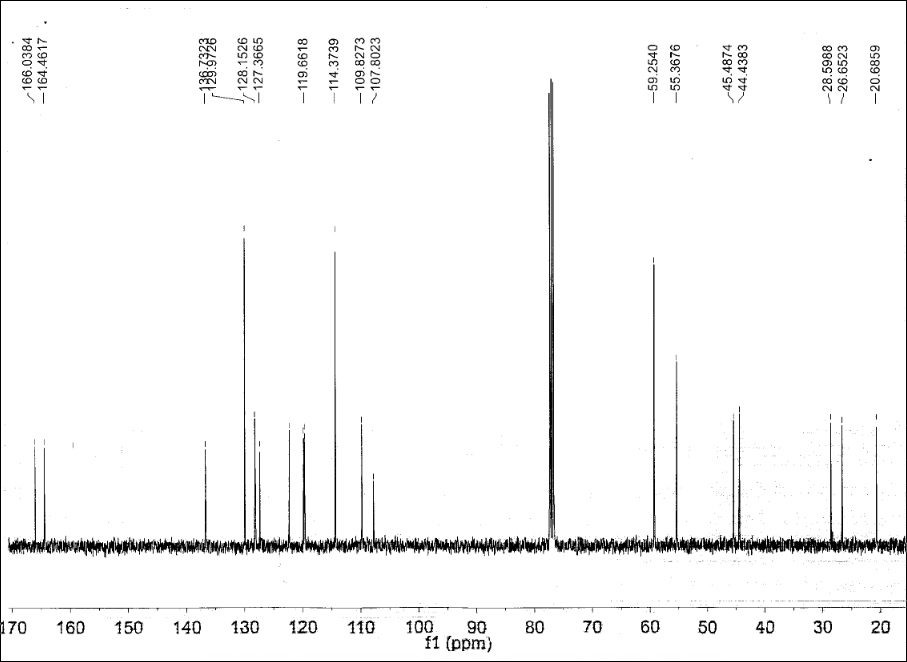

**^13^C-NMR (100 MHz, CDCl_3_) Spectrum of Compound 1b**


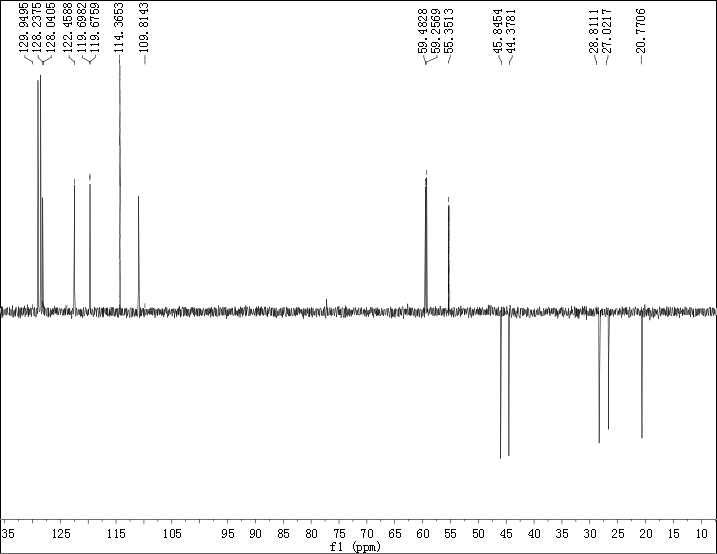


**DEPT-135 Spectrum of Compound 1b**


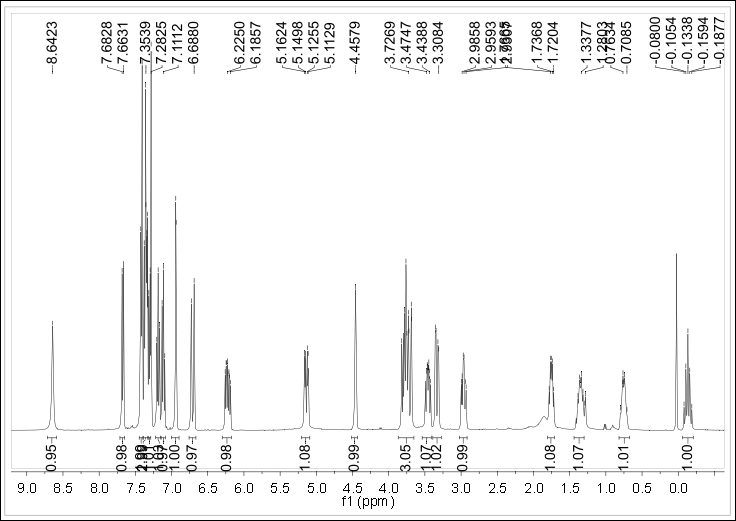

**^1^H-NMR (400 MHz, CDCl_3_) Spectrum of Compound 1c**


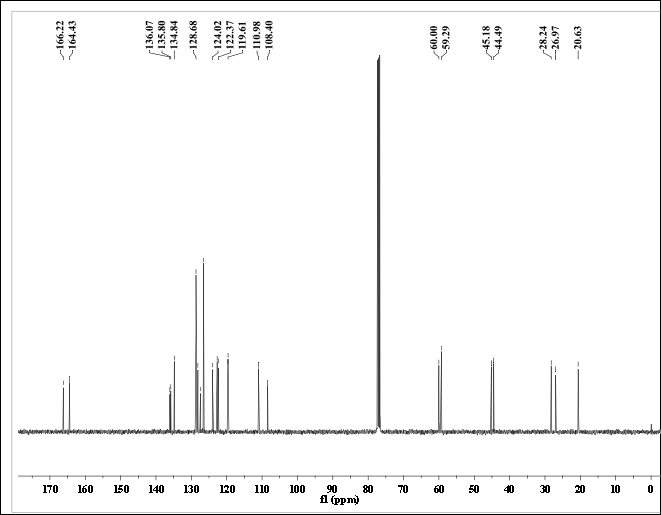

**^13^C-NMR (100 MHz, CDCl_3_) Spectrum of Compound 1c**


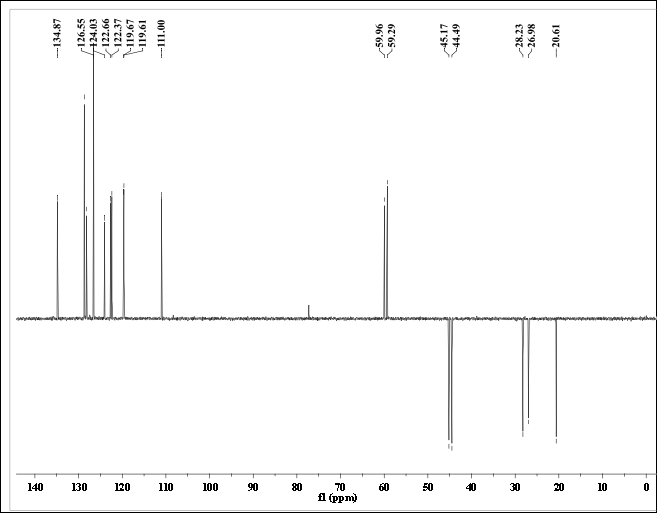


**DEPT-135 Spectrum of Compound 1c**


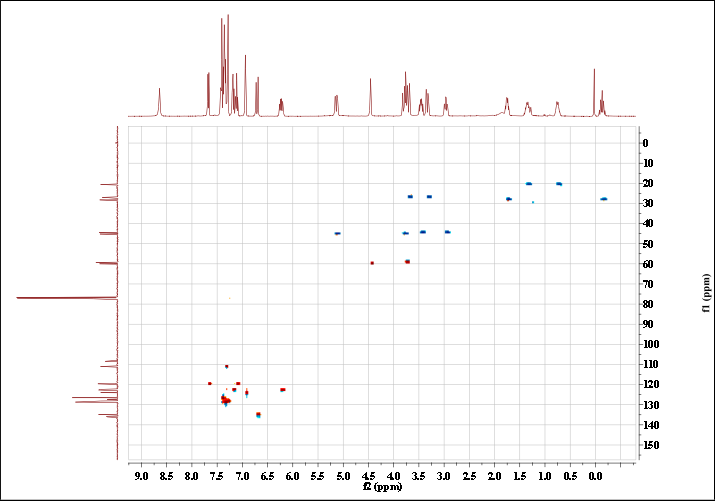
**HSQC of Compound 1c**


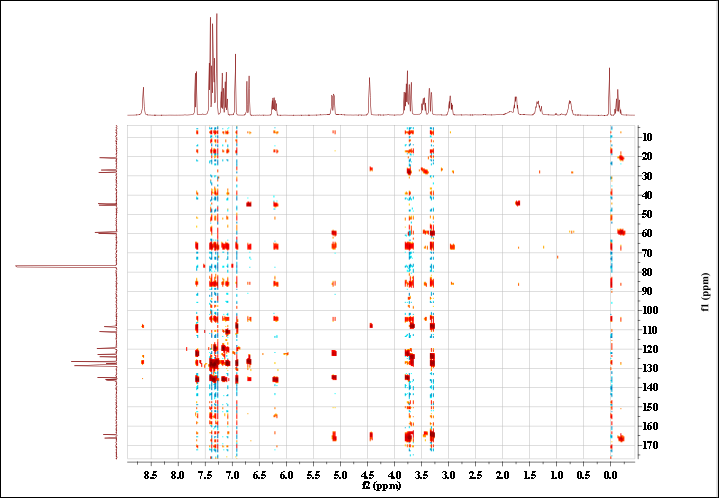


**HMBC of Compound 1c**


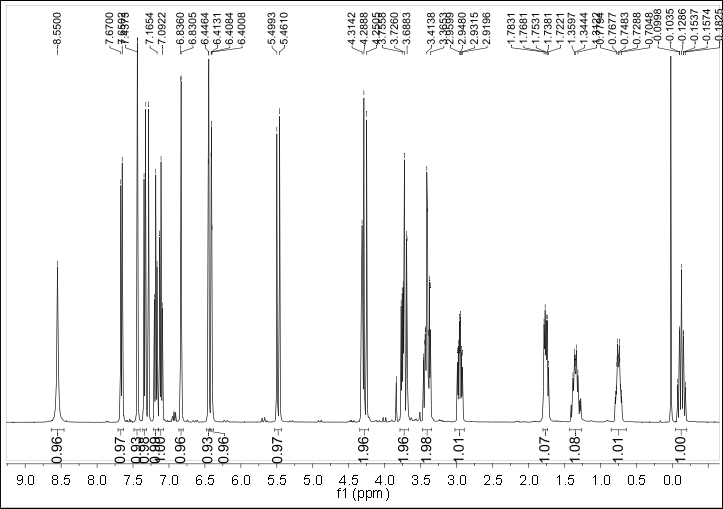

**^1^H-NMR (400 MHz, CDCl_3_) Spectrum of Compound 1d**


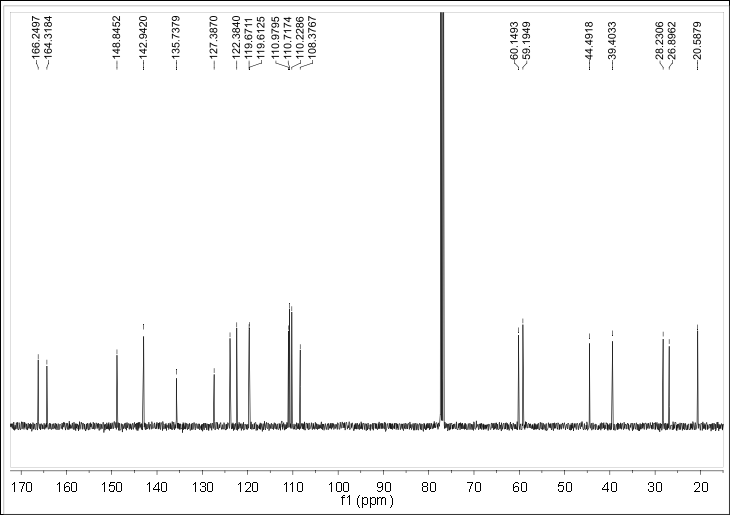

**^13^C-NMR (100 MHz, CDCl_3_) Spectrum of Compound 1d**


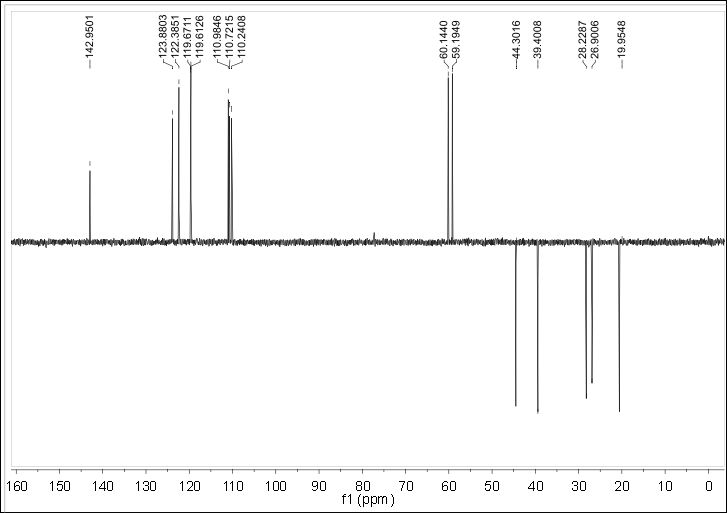


**DEPT-135 Spectrum of Compound 1d**


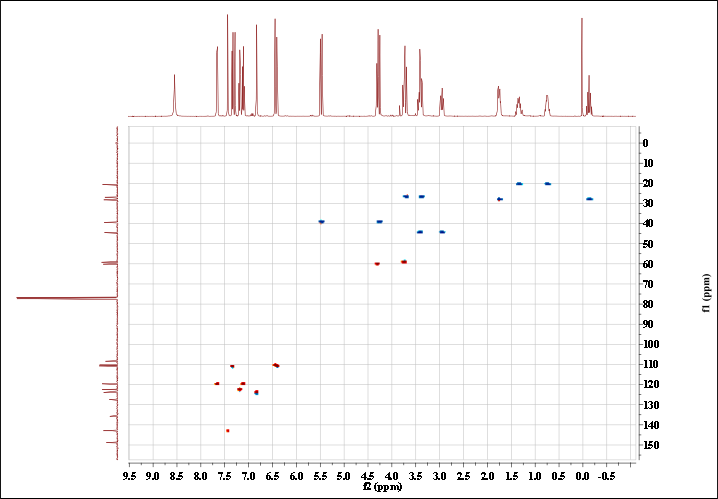


**HSQC of Compound 1d**


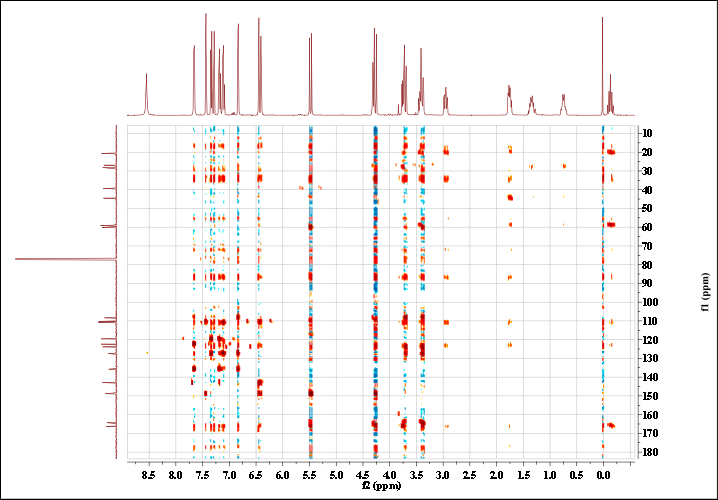


**HMBC of Compound 1d**

**^1^H-NMR (400 MHz, CDCl_3_) Spectrum of Compound 1e**


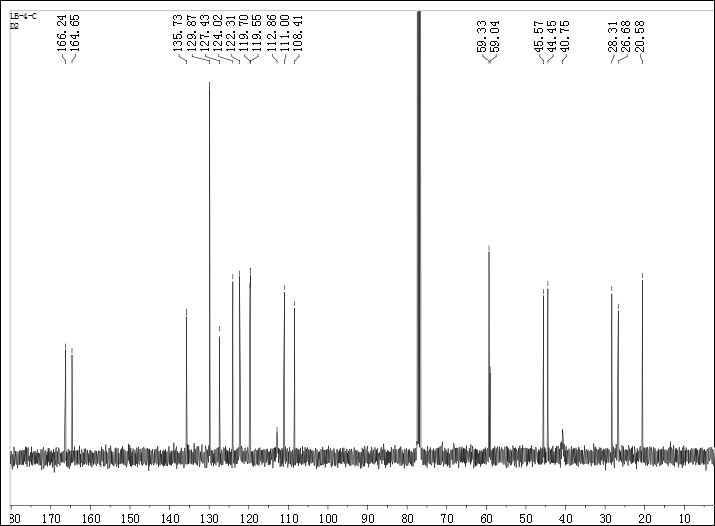

**^13^C-NMR (100 MHz, CDCl_3_) Spectrum of Compound 1e**


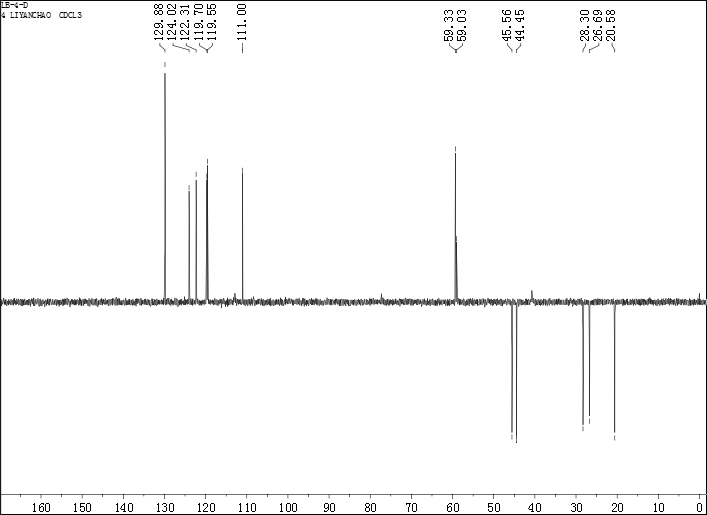


**DEPT-135 Spectrum of Compound 1e**


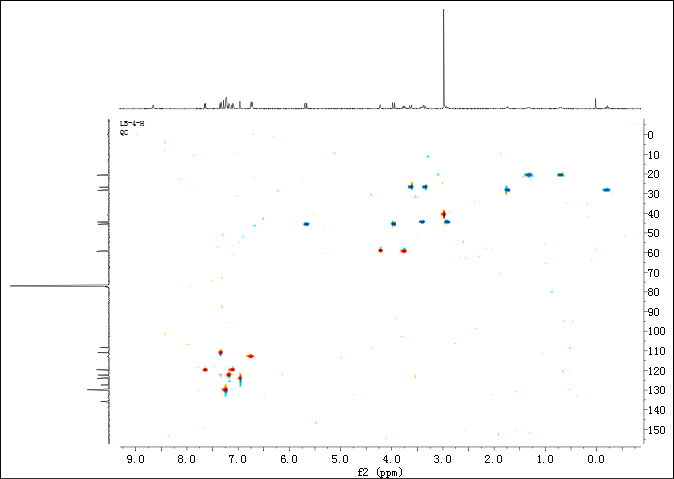


**HSQC of Compound 1e**


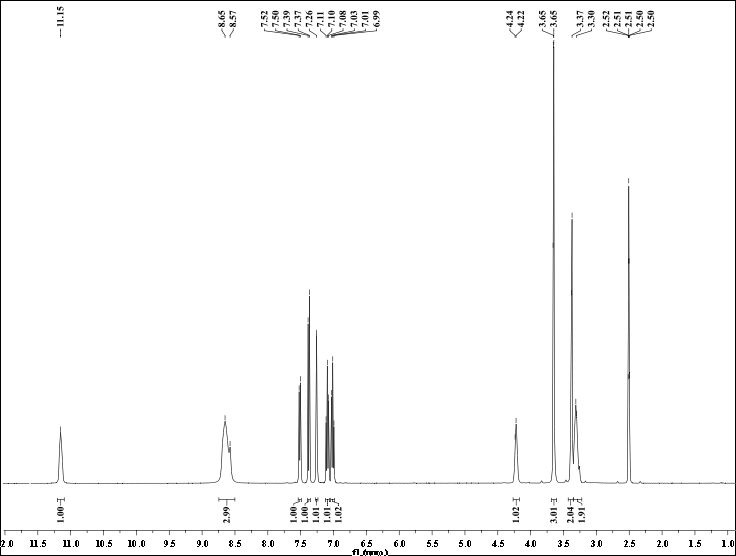

**^1^H-NMR (400 MHz, DMSO) Spectrum of Compound 7**


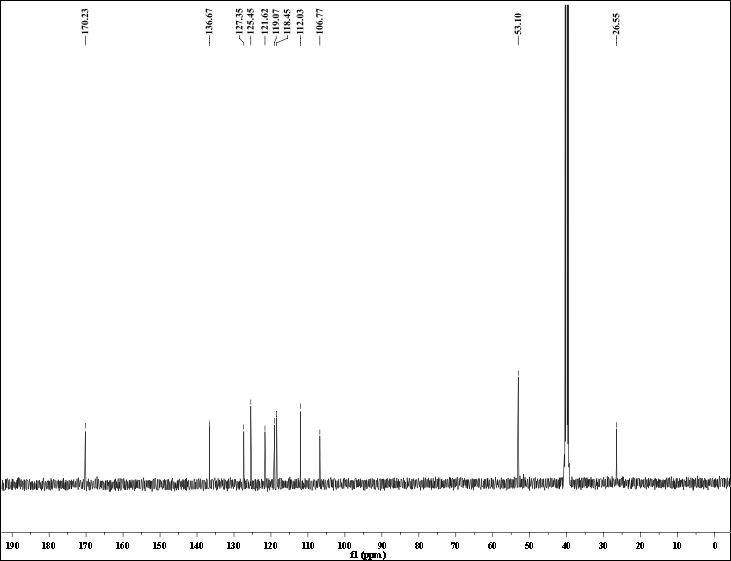

**^13^C-NMR (100 MHz, DMSO) Spectrum of Compound 7**


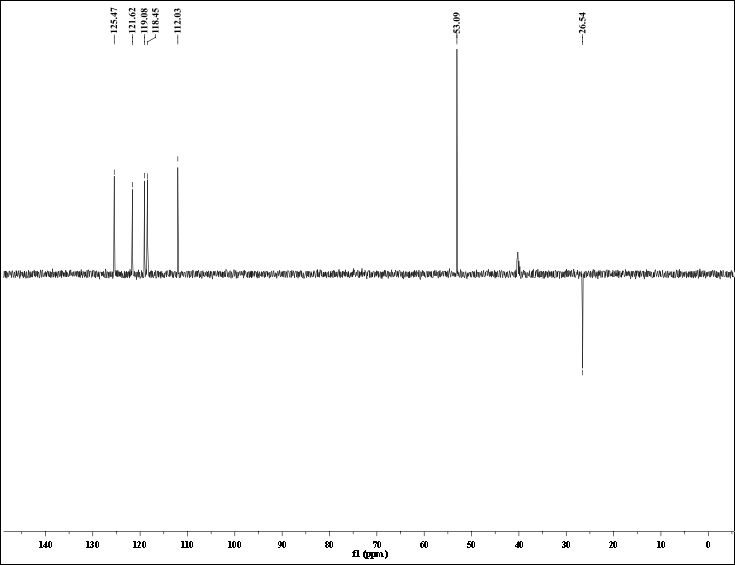


**DEPT-135 Spectrum of Compound 7**


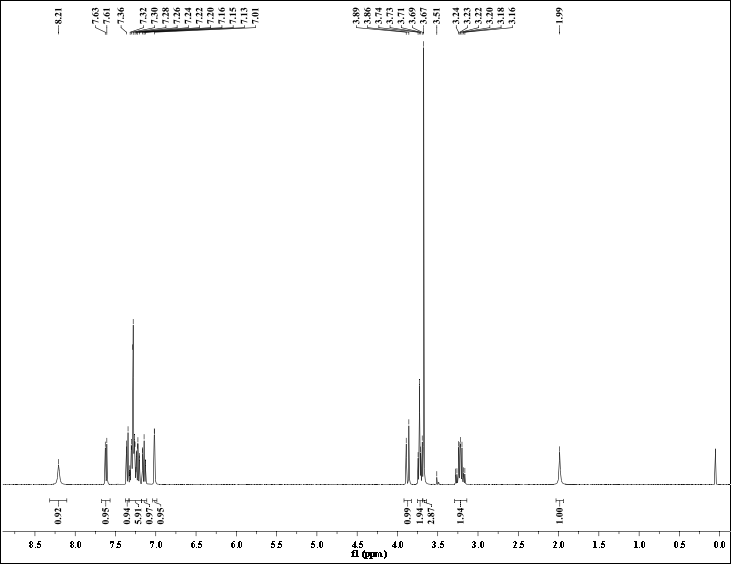

**^1^H-NMR (400 MHz, CDCl_3_) Spectrum of Compound 8a**


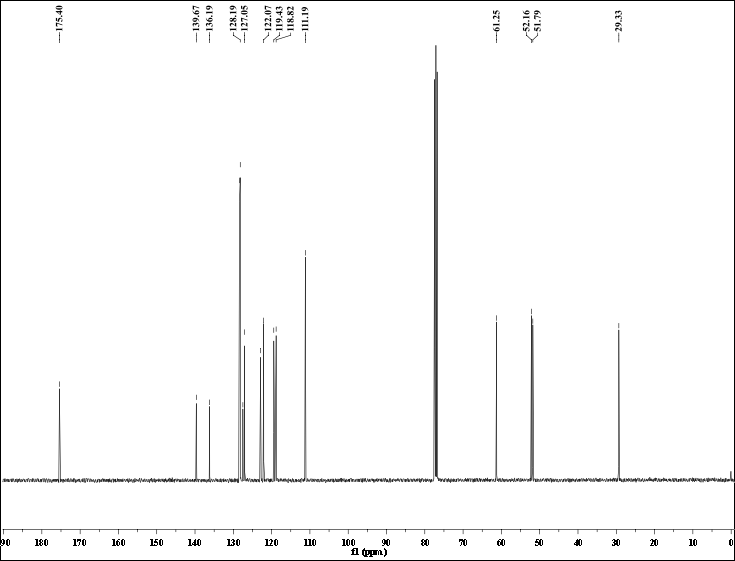

**^13^C-NMR (100 MHz, CDCl_3_) Spectrum of Compound 8a**


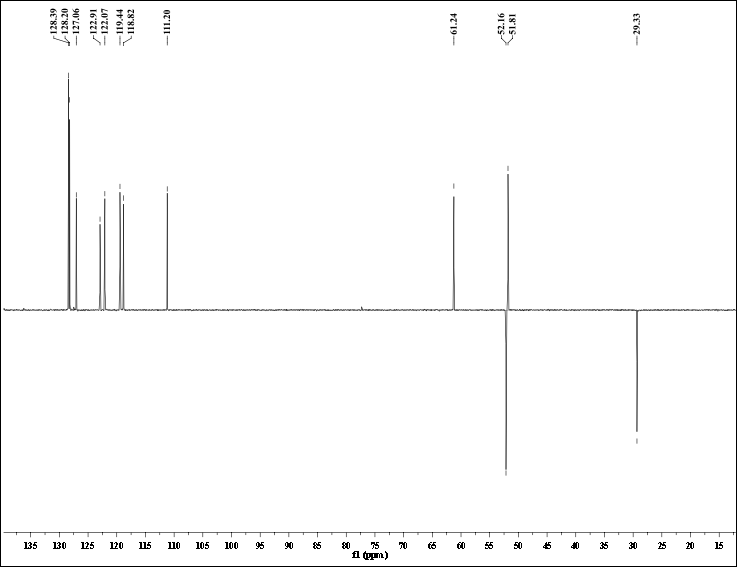


**DEPT-135 Spectrum of Compound 8a**


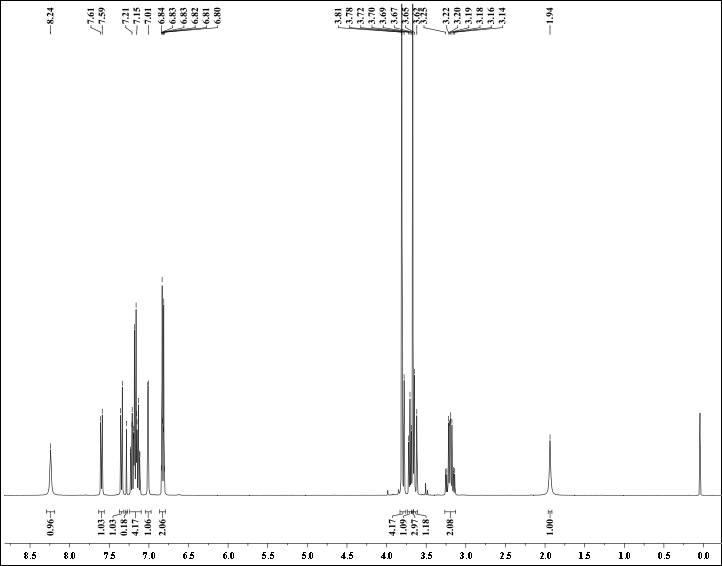

**^1^H-NMR (400 MHz, CDCl_3_) Spectrum of Compound 8b**


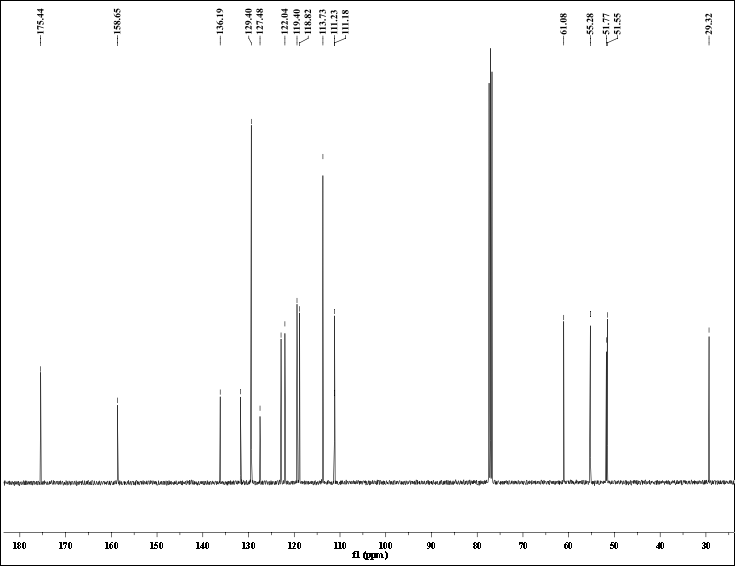

**^13^C-NMR (100 MHz, CDCl_3_) Spectrum of Compound 8b**


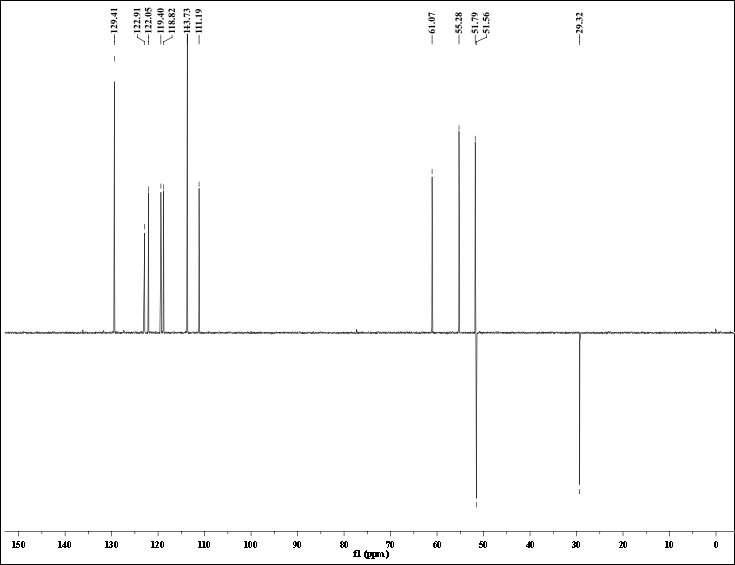


**DEPT-135 Spectrum of Compound 8b**


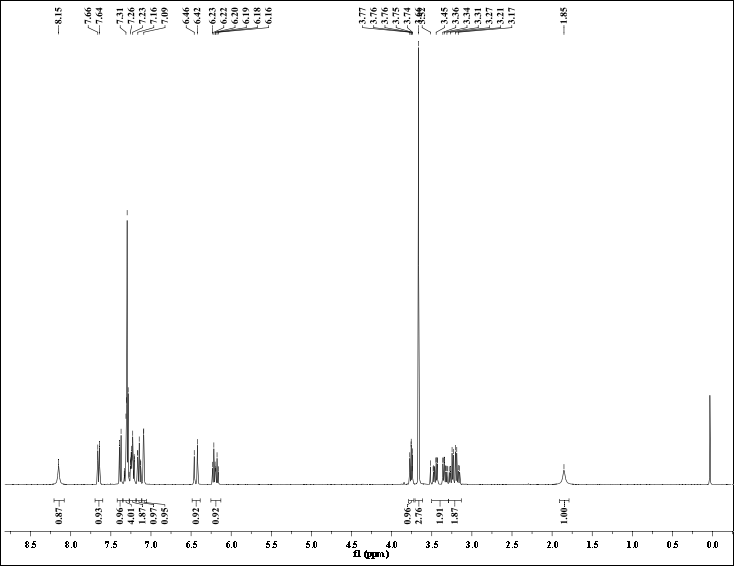

**^1^H-NMR (400 MHz, CDCl_3_) Spectrum of Compound 8c**


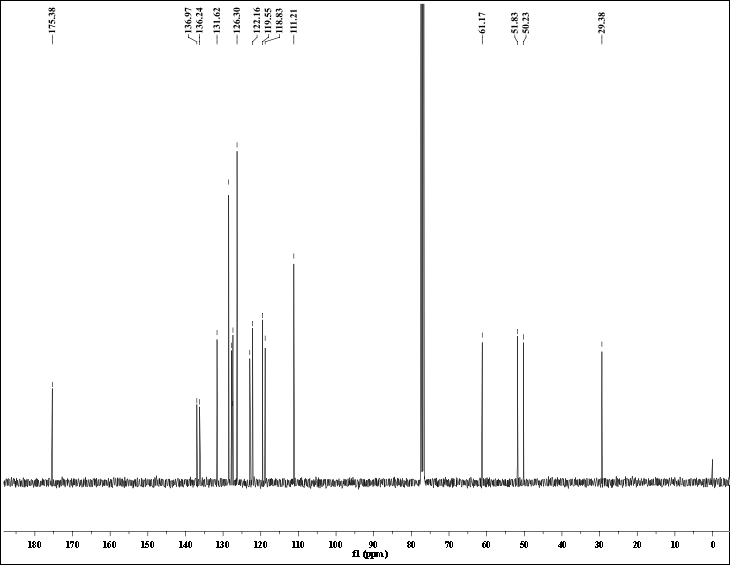

**^13^C-NMR (100 MHz, CDCl_3_) Spectrum of Compound 8c**


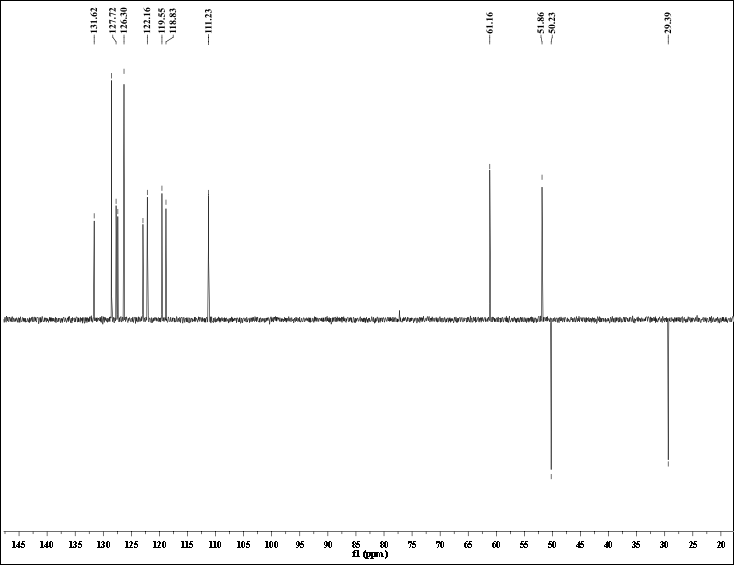


**DEPT-135 Spectrum of Compound 8c**


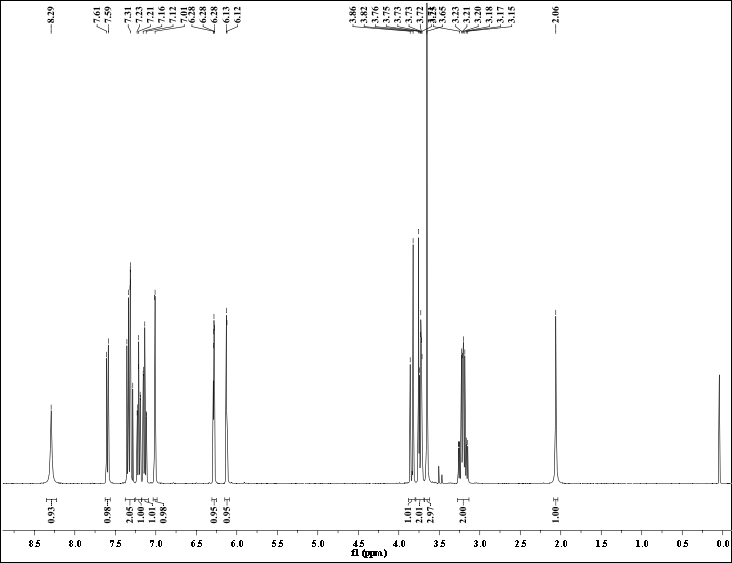

**^1^H-NMR (400 MHz, CDCl_3_) Spectrum of Compound 8d**


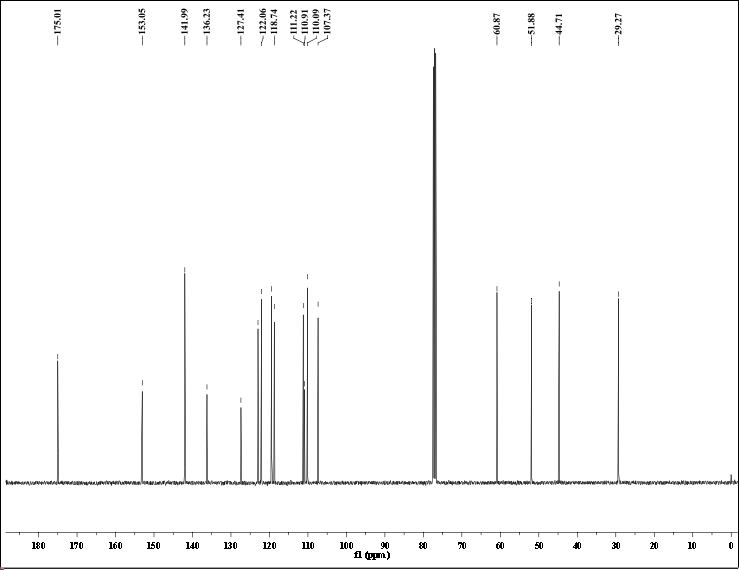

**^13^C-NMR (100 MHz, CDCl_3_) Spectrum of Compound 8d**


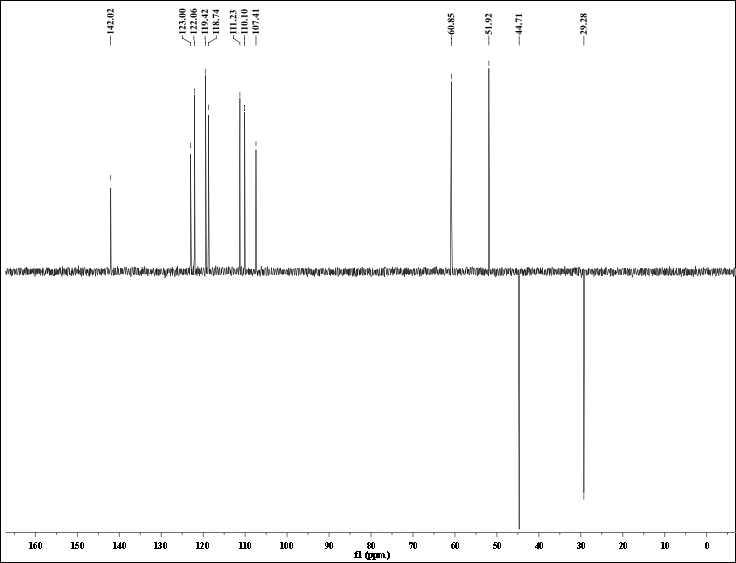


**DEPT-135 Spectrum of Compound 8d**


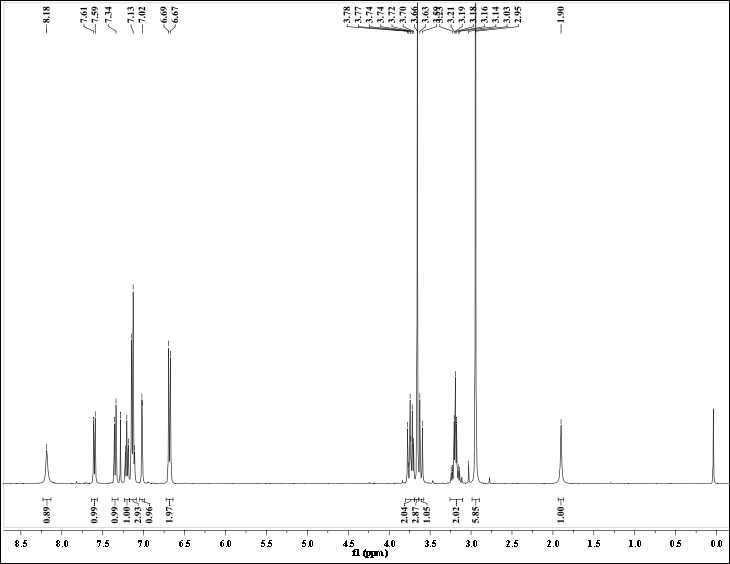

**^1^H-NMR (400 MHz, CDCl_3_) Spectrum of Compound 8e**


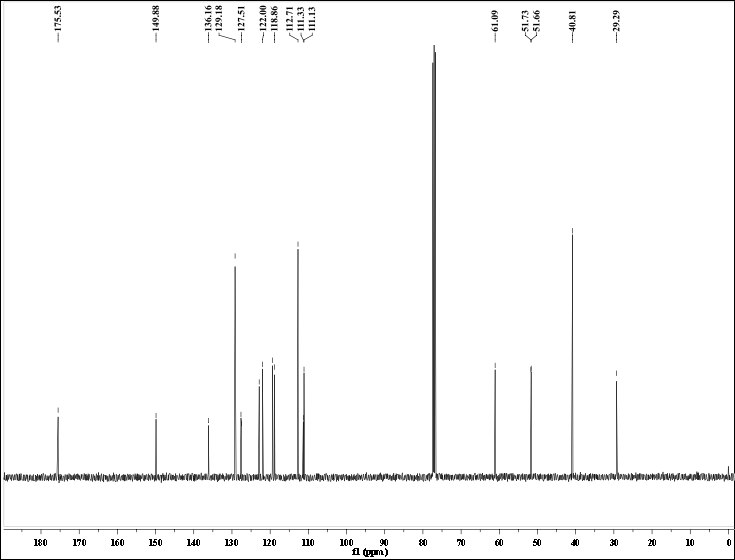

**^13^C-NMR (100 MHz, CDCl_3_) Spectrum of Compound 8e**


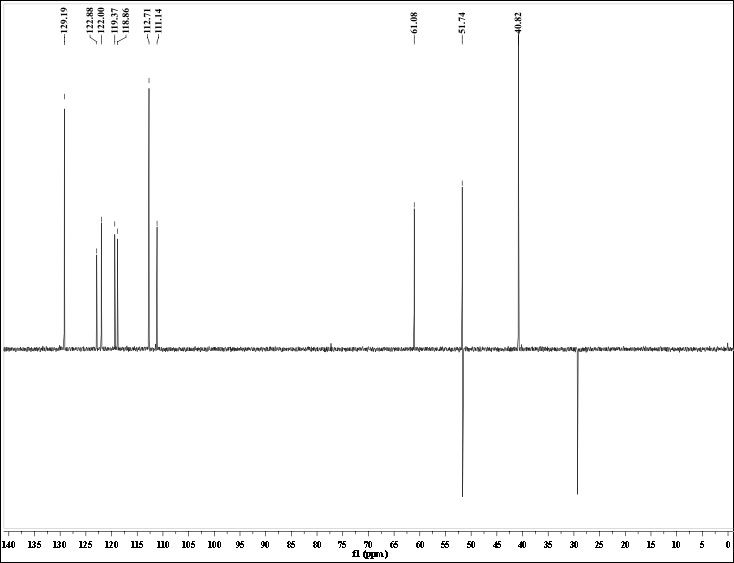


**DEPT-135 Spectrum of Compound 8e**

HRMS of **1b**, **1d** and **1e**


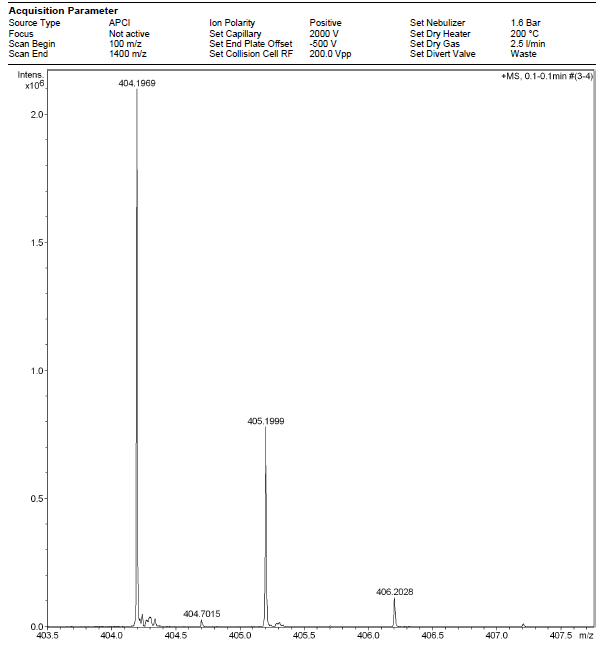


**HRMS of Compound 1b**

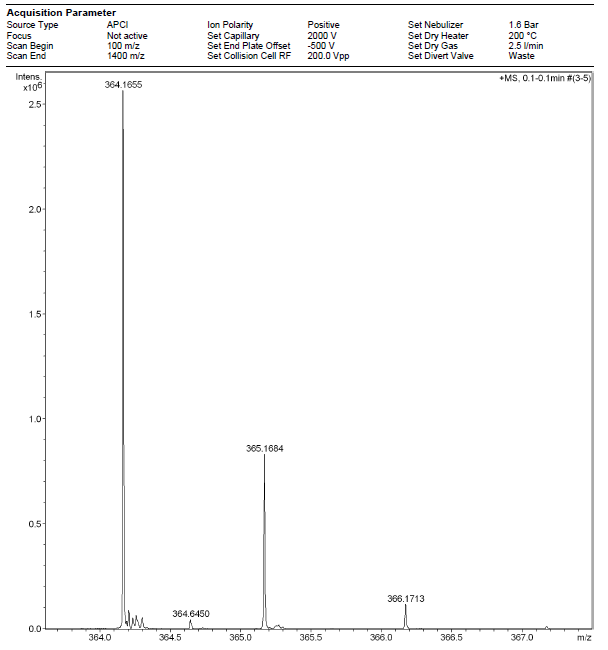


**HRMS of Compound 1d**

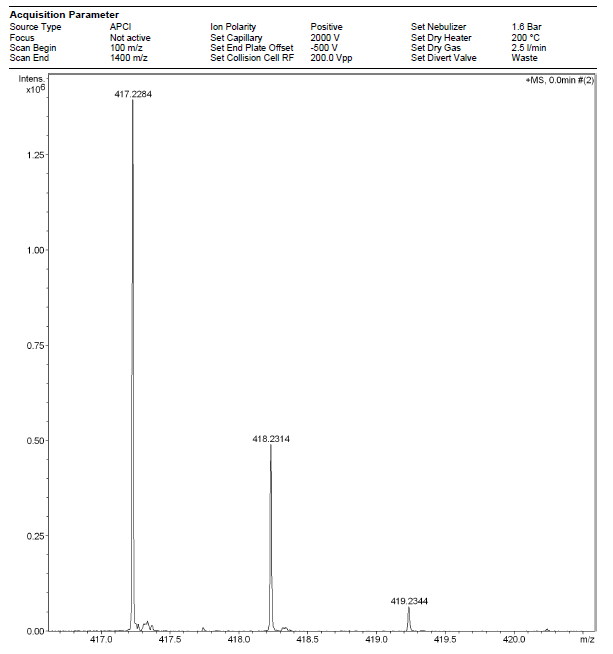


**HRMS of Compound 1e**
